# Functional Roles of PARP2 in Assembling Protein–Protein Complexes Involved in Base Excision DNA Repair

**DOI:** 10.3390/ijms22094679

**Published:** 2021-04-28

**Authors:** Inna Vasil’eva, Nina Moor, Rashid Anarbaev, Mikhail Kutuzov, Olga Lavrik

**Affiliations:** 1 Institute of Chemical Biology and Fundamental Medicine, Siberian Branch of the Russian Academy of Sciences, 630090 Novosibirsk, Russia; iva@niboch.nsc.ru (I.V.); moor@niboch.nsc.ru (N.M.); anarbaev@niboch.nsc.ru (R.A.); kutuzov.mm@mail.ru (M.K.); 2 Department of Natural Sciences, Novosibirsk State University, 630090 Novosibirsk, Russia

**Keywords:** PARP2, PARP1, protein–protein interaction, base excision repair, poly(ADP-ribosyl)ation, fluorescence techniques, dynamic light scattering

## Abstract

Poly(ADP-ribose) polymerase 2 (PARP2) participates in base excision repair (BER) alongside PARP1, but its functions are still under study. Here, we characterize binding affinities of PARP2 for other BER proteins (PARP1, APE1, Polβ, and XRCC1) and oligomerization states of the homo- and hetero-associated complexes using fluorescence-based and light scattering techniques. To compare PARP2 and PARP1 in the efficiency of PAR synthesis, in the absence and presence of protein partners, the size of PARP2 PARylated in various reaction conditions was measured. Unlike PARP1, PARP2 forms more dynamic complexes with common protein partners, and their stability is effectively modulated by DNA intermediates. Apparent binding affinity constants determined for homo- and hetero-oligomerized PARP1 and PARP2 provide evidence that the major form of PARP2 at excessive PARP1 level is their heterocomplex. Autoregulation of PAR elongation at high PARP and NAD^+^ concentrations is stronger for PARP2 than for PARP1, and the activity of PARP2 is more effectively inhibited by XRCC1. Moreover, the activity of both PARP1 and PARP2 is suppressed upon their heteroPARylation. Taken together, our findings suggest that PARP2 can function differently in BER, promoting XRCC1-dependent repair (similarly to PARP1) or an alternative XRCC1-independent mechanism via hetero-oligomerization with PARP1.

## 1. Introduction

Base excision repair (BER) in mammalian cells is an efficient and complex process developed to detect and repair damaged bases and apurinic/apyrimidinic (AP) sites in DNA. Repair of single-strand breaks (SSB) involves enzymes and factors of the BER system and is generally considered a separate pathway of this process [1]. Many proteins and factors participate in this process via their enzymatic and regulatory/coordinating functions [2,3]. One of the regulation mechanisms of BER is the poly(ADP-ribosyl)ation (PARylation) [4,5,6,7,8,9,10,11]. The synthesis of an ADP-ribose polymer (PAR) is catalysed by poly(ADP-ribose) polymerases, specific enzymes capable of modifying themselves and target proteins. Among the PARP superfamily, poly(ADP-ribose) polymerase 1 (PARP1) is the major coordinating protein of BER [6,9,10]. PARP1 serves as a sensor of the DNA lesions usually induced by ionizing irradiation and oxidative stress and signals to recruit the appropriate proteins to the sites of damage [1,12]. Poly(ADP-ribose) polymerase 2 (PARP2), the second DNA-dependent member of the PARP superfamily, is another component of a functional BER complex in vivo [13]. In human cells, the majority of PARP activity is exerted by PARP1 (85–90%) and by PARP2 (10–15%) [9]. It is known that cells can survive without either PARP1 or PARP2, but a double knockout is embryonic lethal [14]; these data demonstrate overlapping of crucial functions of PARPs 1 and 2 in maintaining genome stability and cell survival. PARP2 differs substantially from PARP1 in the domain architecture, but shares significant structural homology with the catalytic domain of PARP1 [5,9,15]. The participation of PARP2 in BER has been proposed to be mediated by its interaction with PARP1 [13]. Similar to PARP1, PARP2 can interact with X-ray repair cross-complementing protein 1 (XRCC1) and the main BER enzymes DNA polymerase β (Polβ) and DNA ligase IIIα (LigIIIα) on the DNA damage sites and can regulate repair processes through its direct interaction with proteins and DNA intermediates [13,16,17,18,19]. Based on difference between PARP1 and PARP2 in recruitment kinetics to DNA-damaged sites and selective activation of PARP2 by 5′-phosphorylated DNA intermediates, PARP2 has been proposed to act at later stages of the repair process, in contrast to PARP1 acting at an earlier stage [20,21]. Nevertheless, despite convincing evidence supporting the main contribution of PARP1 to regulation of BER and repair of SSBs, the specific function of PARP2 in these processes is still under detailed study [8,9,19,22].

In our previous study, we detected and characterized the interaction of PARP1 with functionally different key protein players of BER, AP endonuclease 1 (APE1), Polβ, and XRCC1, by using fluorescence-based and light-scattering techniques [23,24]. Here, we performed similar biophysical studies for PARP2 protein and its complexes. The relative binding affinities of PARP2 for the main BER proteins and oligomerization states of the homo- and hetero-associated complexes were determined for the first time. The size of the PARP2 molecule covalently bound with PAR polymers synthesized upon the automodification reaction in the absence and presence of various key BER proteins was measured and compared with results obtained previously for PARylated PARP1 [24]. To visualize the intermolecular associates detected by DLS for PARylated PARP1/PARP2 and their protein mixtures, we applied fluorescent detection by Celena S Digital Imaging, using proteins labelled with distinct fluorophores.

## 2. Results

### 2.1. Detection and Quantification of PARP2–Protein Interactions by Fluorescence Titration and FRET Experiments

Interaction of PARP2 with the main BER proteins was studied by the fluorescent titration method using fluorophores with different spectral characteristics and hydrophobic properties. 5,6-Carboxyfluorescein- (FAM-), 5-Alexa Fluor 488- (AF-), sulfo-Cyanine 3- (Cy3-) and sulfo-Cyanine 5- (Cy5-) labelled proteins (PARP2, APE1, Polβ, PARP1, and XRCC1) were prepared using succinimidyl esters of respective fluorophores. The reaction was performed at pH 7.0 to label proteins preferentially at the N-terminal amino group [25]. In each case, the reaction conditions were optimized by varying the protein and reagent concentrations in order to obtain an active protein containing no more than one fluorescent label per protein molecule. Characteristics of the labelled proteins used in this study are detailed in Supplementary Table S1.

**Figure 1:**
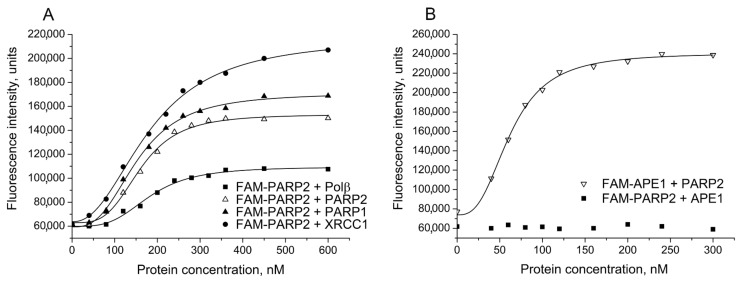
Detection of PARP2 complexes with various BER proteins by fluorescence titration of FAM-PARP2 or FAM-APE1. The FAM-labelled protein (40 nM) was excited at 482 nm in the absence or presence of increasing concentrations of the binding partner indicated, and the relative fluorescence intensities were monitored at 530 nm. Typical titration curves representing binding of FAM-PARP2 to Polβ, PARP2, PARP1, and XRCC1 (**A**), and complex formation between FAM-APE1 and PARP2 (**B**) show the best fits of the four-parameter equation (with *R*^2^ values exceeding 0.98); no detectable change in the fluorescence intensity of FAM-PARP2 in the presence of APE1 is demonstrated (**B**).

Initial studies were performed by using FAM-labelled PARP2 (or FAM-labelled partner protein) to determine relative affinities of PARP2 for other BER proteins under the experimental conditions developed previously for detection of various BER protein–protein complexes, including those of PARP1 [23]. Interaction between PARP2 and protein partners was studied by monitoring the change in the fluorescence intensity of FAM-PARP2 in the presence of unlabelled proteins (PARP2, XRCC1, PARP1, Polβ or APE1) added at increasing concentrations. Typical curves obtained for FAM-PARP2 titration with unlabelled PARP2, PARP1, Polβ, and XRCC1 are shown in Figure 1A. A substantial increase in fluorescence intensity of FAM-PARP2 (from 1.8- to 3.5-fold) in the presence of saturating concentrations of the most protein partners was detected, indicating that the local environment of the fluorophore changed upon protein–protein association. The addition of APE1 had no effect on FAM-PARP2 fluorescence intensity, while a significant increase in the FAM-APE1 fluorescence induced by the interaction with PARP2 was detected (Figure 1B). The binding parameters of PARP2 interaction with proteins presented in Table 1 (EC_50_) are apparent equilibrium dissociation constants of the complexes. The EC_50_ values determined for PARP2 complexes with Polβ and XRCC1 are practically identical to each other and slightly (from 1.2- to 1.3-fold) exceed those characterizing self-association of PARP2 and its hetero-oligomerization with PARP1, indicating that PARP2 can interact with homologous and functionally different heterologous proteins with closely similar affinities. The affinity of PARP2 for APE1 was determined to be 3-fold higher than the affinity of PARP2 for Polβ.

**Figure 2:**
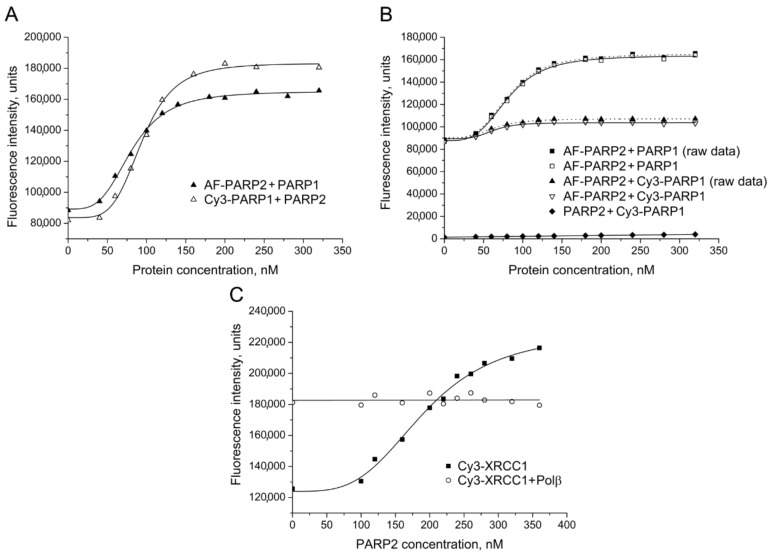
Detection of PARP2 interaction with PARP1 and XRCC1 by using AF and Cy3 as fluorophores. (**A**) Fluorescence titration of AF-PARP2 and Cy3-PARP1 (40 nM) with unlabelled PARP1 and PARP2, respectively. (**B**) FRET experiments were performed by titration of AF-PARP2 (40 nM) with unlabelled and Cy3-labelled PARP1. The efficiency of FRET was determined from the corrected data (open symbols) obtained from the raw data (filled squares and triangles) by subtraction of the background or Cy3 fluorescence intensities that were measured in control series by titration of unlabelled PARP2 with Cy3-labelled PARP1 (filled diamonds). (**C**) Fluorescence titration of Cy3-XRCC1 (40 nM) in the absence and presence of Polβ (200 nM) with PARP2 protein. Curves show the best fits of the four-parameter equation (with *R*^2^ values exceeding 0.97).

Unfortunately, the fluorescence signal of FAM-labelled PARP2 was unstable during storage probably due to nonspecific aggregation. Sulfonated dyes with different spectral properties, sulfo-cyanines 3 (Cy3) and 5 (Cy5), and the rhodamine dye Alexa Fluor 488 (AF488, 5-isomer) were additionally tested as fluorescent labels. The presence of sulfonate groups in the dye molecules improves solubility of their succinimidyl esters and decreases aggregation of labelled proteins. Furthermore, Cy3 and Cy5 are characterized by photostability, high extinction coefficients, and high quantum yields, enabling sensitive detection [26]. The interaction between PARP2 and PARP1 was detected and quantified by titration of either AF-PARP2 with unlabelled PARP1 or Cy3-PARP1 with unlabelled PARP2 (Figure 2A,B). However, the addition of Polβ, APE1 or XRCC1 produced no detectable effect on the AF-PARP2 fluorescence intensity (Figure S1). We were also unable to detect a change in the fluorescence intensity of Cy3-Polβ in the presence of PARP2. The EC_50_ values determined for AF-PARP2–PARP1 and Cy3-PARP1–PARP2 pairs are practically identical to each other but substantially (~1.9-fold) lower than the respective value determined for the FAM-PARP2–PARP1 pair (Table 1). Thus, presence of the negatively charged sulfonate groups in a fluorophore attached to either PARP2 or PARP1 influences the formation of contacts between these proteins. On the contrary, practically identical EC_50_ values were determined for the PARP2–XRCC1 complex by using either FAM-PARP2 or Cy3-XRCC1 as the labelled partner (Figure 1A and Figure 2C; Table 1), suggesting that the labelling of either partner with chemically different fluorophores did not disturb the protein–protein interaction. Notably, the fluorescence signal of Cy3-XRCC1 premixed with an excessive concentration of Polβ was unchanged upon addition of increasing concentrations of PARP2 (Figure 2C). The initial fluorescence intensity of Cy3-XRCC1 was significantly higher in the presence of Polβ than in its absence due to formation of the respective binary complex shown previously to be the most stable in the BER system [23]. The data suggest that the local environment of the fluorophore attached to the N-terminal domain of XRCC1 is fully created by strong interaction with Polβ and is unchangeable upon formation of a possible ternary XRCC1 complex with Polβ and PARP2. The XRCC1 binding sites for Polβ and PARP2 are localized on the distinct structural domains (N-terminal and BRCTa, respectively [2], enabling formation of the ternary complex.

Direct physical interaction of PARP2 with PARP1 was further confirmed by FRET, using AF-labelled PARP2 and Cy3-labelled PARP1 as a donor–acceptor pair. The fluorescence was excited and monitored at the excitation and emission maxima of 5-Alexa Fluor 488. To correct for the fluorescence signal produced by the acceptor, unlabelled PARP2 was titrated with Cy3-labelled PARP1 in the control experiment. The corrected data were plotted to obtain the corrected binding curve (fitted to the four-parameter equation) (Figure 2B). The fluorescence intensity of AF-PARP2 increased less in the presence of Cy3-PARP1 than in the presence of unlabelled PARP1, indicating that the donor-labelled and acceptor-labelled proteins participate in FRET. The efficiency of FRET was calculated from the fractional decrease of the fluorescence intensity of the donor due to the presence of the acceptor: E = 1 − F_da_/F_d_, where F_da_ and F_d_ are the AF-PARP2 fluorescence intensities measured in the presence of identical concentrations of Cy3-PARP1 (the corrected values) or PARP1, respectively.

The influence of model DNAs mimicking intermediates of the BER process on the interaction of PARP2 with proteins was studied. The titration experiments for FAM-PARP2 with three various unlabelled proteins were performed in the absence and presence of the DNA duplex with a single-nucleotide gap (gap-DNA), a canonical substrate of Polβ, added at an excessive concentration (Figure S2). The binding affinity of FAM-PARP2 for Polβ, XRCC1, and PARP2 increased to a different extent in the presence of the key DNA intermediate of BER, with the highest effect having been detected for the complex with Polβ (Table 2). DNA duplex with a single-strand break (nick-DNA) and undamaged (free of SSB) DNA duplex (ds-DNA) were comparatively tested for modulating effects on the quantitative characteristics of the PARP2–PARP1 interaction (Figure S4). The two DNAs were shown to differ in their affinity for PARP2 and produce quite distinct effects on the efficiency of FRET (4% decrease and 8% increase in the presence of nick-DNA and ds-DNA, respectively), that reflected the DNA-induced structural rearrangement of the PARP2–PARP1 complex (Table 2). The affinity of AF-PARP2 for PARP1 increased substantially (by 1.8-fold) in the presence of nick-DNA, but decreased to the same extent in the presence of ds-DNA, indicating specific stabilization of the PARP2-PARP1 interaction by the SSB-containing DNA.

### 2.2. DLS Study of Oligomerization States of PARP2 and Its Hetero-Associated Complexes with BER Proteins

**Figure 3:**
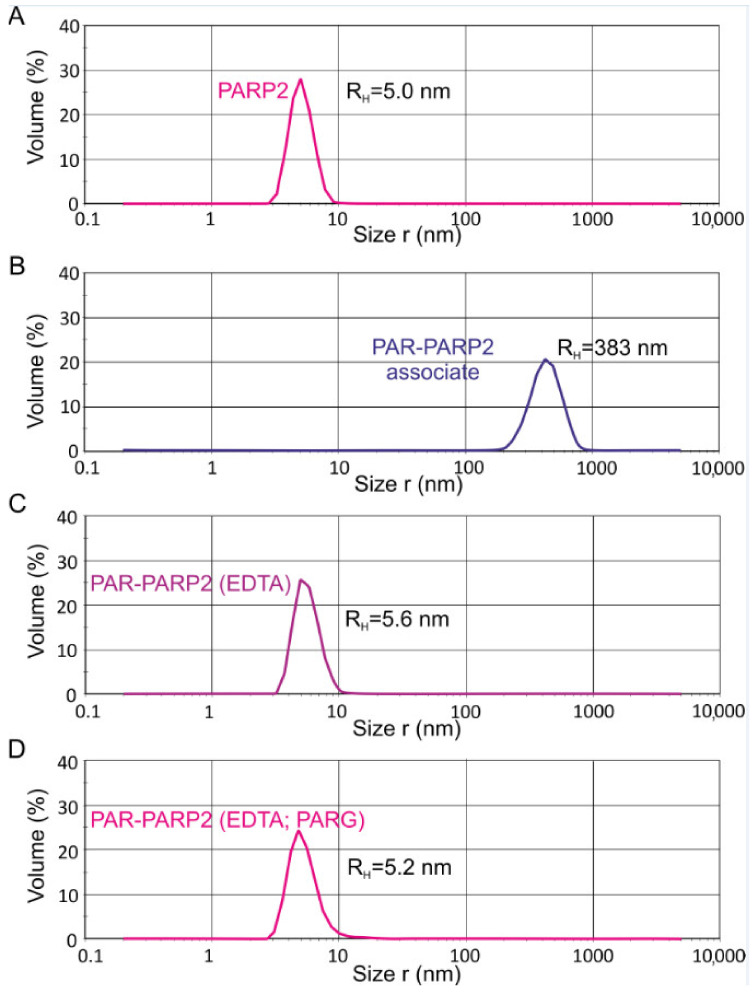
Volume-weighted size distribution profiles for PARP2 protein (**A**) and its PARylated (PAR-PARP2) form (**B**–**D**). The R_H_ values of PAR-PARP2 were measured directly after 15-min incubation with NAD^+^ and DNA (when the size growth reached the plateau) (**B**) and after the addition of EDTA (**C**) and treatment with PARG (**D**).

The applicability of dynamic light-scattering (DLS) technique for determination of hydrodynamic sizes of BER proteins and protein–protein complexes to estimate their oligomeric state under true equilibrium conditions in solution was demonstrated for the first time in our previous work [24]. Similar DLS experiments, complemented with a chemical cross-linking study, were conducted here for PARP2 and its binary complexes with other BER proteins. All measurements were performed and analysed using a Zetasizer Nano ZS instrument and software. Assuming particle sphericity and considering mathematic approximation to the non-monomodal distribution, the particle size distributions were fitted to yield the hydrodynamic radius (R_H_). All three types of particle size distributions (intensity, volume, and number weighted) were used for analysis. A volume-weighted distribution was applied as more relevant for estimation of the oligomeric state, because in this type of distribution, the contribution of each particle is related to the volume/mass of the particle that in accordance with Rayleigh approximation is proportional to R^3^. A typical volume-weighted size distribution profile obtained for PARP2 as an example is presented in Figure 3A. The experimentally determined volume-weighted size distribution R_H_ value for PARP2 slightly exceeded the theoretical value for the homodimer (5.0 vs. 4.67 nm) and was significantly lower than the respective value predicted for the homotetramer (5.0 vs. 6.28 nm) (Table 3). We found that the amount of species different from the homodimer was minor: the R_H_ values determined from three size distributions varied within the experimental error (from 4.6 ± 0.3 to 5.4 ± 0.4 nm; Table S2). Thus, we propose that the main homo-oligomeric form of PARP2 in solution exists as a dimer.

DLS measurements were performed for the equimolar mixtures of PARP2 with various BER proteins (Polβ, APE1, XRCC1, and PARP1) characterized as the PARP2-binding partners. Values of the apparent equilibrium dissociation constants determined for the respective homo- and hetero-oligomeric complexes here (Table 1) and previously [23] are comparable for the most complexes, suggesting their coexistence at almost equal proportion in the equimolar mixtures of high concentration (used in the DLS experiments). Indeed, the glutaraldehyde cross-linking experiments detailed in Supplementary Results revealed that cross-linked products specific for individual proteins and their hetero-associated complexes were formed in the cross-linked equimolar mixtures of the protein pairs (Figure S5A,B).

The R_H_ value determined from the volume-weighted size distribution for the binary mixture of PARP2 with Polβ exceeded the respective value for the individual PARP2 (5.6 vs. 5.0) and was slightly lower than the theoretical value for the dimer-of-heterodimers (heterotetramer) (5.74) as a result of contribution of the homo-oligomerized small protein partner–Polβ (3.5) (Table 3). Thus, the most probable oligomeric state of the PARP2–Polβ complex is heterotetrameric. For the binary mixture of PARP2 with APE1, another small protein closely similar to Polβ in size, the experimental R_H_ value determined from volume-weighted size distribution to be between the theoretical values for the heterodimer and heterotetramer was lower than the respective value for the PARP2 mixture with Polβ (4.9 vs. 5.6) (Table 3). Based on this observation, we suggest that the PARP2–APE1 complex exists as an equilibrium mixture of heterodimeric and heterotetrameric forms.

The R_H_ value determined from the volume-weighted size distribution for the binary mixture of PARP2 with XRCC1 (7.6) substantially exceeded the respective values for the PARP2 homo-oligomer (5.0) and XRCC1 homo-oligomer (5.7) (Table 3) and was significantly higher than the theoretical value for the heterotetramer (AB)_2_ (6.44). The presence of self-associated proteins in the PARP2–XRCC1 mixture detected by the cross-linking experiments (Supplementary Results; Figure S5A) evidently contributed to the measurements, suggesting that the true hydrodynamic radius of the PARP2–XRCC1 complex was higher than the R_H_ value determined for the mixture. The higher experimental R_H_ value relative to the theoretical one may indicate that the shape of the PARP2–XRCC1 complex deviates from a spherical shape. The non-globular shape of the XRCC1 homo-dimer proposed to be a prolate ellipsoid [27] can be responsible for the extended conformation of its heterocomplex with PARP2. The R_H_ value determined from the volume-weighted size distribution for the binary mixture of PARP2 with the largest binding partner PARP1 (7.8) was comparable to the respective value for the PARP1 homo-oligomer (8.0) and exceeded the theoretical R_H_ value for the heterotetramer (AB)_2_ (7.28) (Table 3). In the previous DLS study, we proposed that the main homo-oligomeric form of PARP1 in solution exists as an extended dimer capable of forming higher order homo-oligomers [24]. Taking all the data together, we conclude that PARP2 forms mainly heterotetrameric complexes with the non-globular PARP1 and XRCC1 proteins.

### 2.3. DLS Detection of Association of Poly(ADP-ribosyl)ated PARP2 in Solution

In our previous study, we applied, for the first time, the DLS technique to measure the hydrodynamic radius of PARP1 covalently bound with PAR polymers synthesized upon the automodification reaction in solution [24]. Similar measurements under identical reaction conditions were performed here for PARP2. We used the same 32-mer double-stranded DNA intermediate containing a one-nucleotide gap (gap-DNA) for activation of PARP1 and PARP2. Real-time measurements of the R_H_ value were performed every 3 min after initiation of the reaction by addition of NAD^+^ substrate, Mg^2+^ and gap-DNA to the protein sample. The magnesium ion coordinates intermolecular bonds with polyphosphate ligands [28] and stabilizes the huge associate detected for PARylated PARP1, that can be disrupted by addition of EDTA, thus enabling us to determine the real size of the autoPARylated enzyme [24]. Typical volume-weighted size distribution profiles obtained for PARP2 upon the automodification reaction are presented in Figure 3, and their comparison with the respective profiles for PARP1 is shown in Figure S6. The R_H_ values presented in Table 4 were determined as the plateau values after 15-min incubation (Figure S7), without or with treatment with EDTA and PAR glycohydrolase (PARG). PARG catalyses degradation of PAR due to cleavage of the ribose-ribose bonds between the ADP-ribose units, releasing the mono(ADP-ribose) attached to a protein [29,30]. The R_H_ value measured before initiating the PARylation reaction was undertaken as a null point. Control measurements performed for the PARP2 mixture with activating DNA (in the absence and presence of other proteins), before addition of NAD^+^, revealed no change in the hydrodynamic radius due to the DNA presence. The R_H_ value determined for PARylated PARP2 associate was 2.6 times lower than that for PAR-PARP1 (Table 4 and Table S3, Figure S7). A comparative gel electrophoresis analysis of PARylated PARP2 and PARP1 and chain length of PAR polymers detached from the proteins by alkaline hydrolysis (Figure S8) revealed that short PAR chains (≤10-mer) constituted the most part of PAR polymers synthesized by PARP2, in contrast to highly elongated and branched polymers synthesized predominately upon the PARP1 automodification. Thus, in the reaction conditions used, PARP2 catalyses less efficiently the elongation and branching of PAR polymers than PARP1 does.

After addition of the EDTA excess, we observed a strong decrease in the R_H_ value of PAR-PARP2 (Figure 3C and Table 4). The real hydrodynamic size of PAR-modified PARP2 was found to be ~4 times smaller than the respective value for PAR-PARP1 (Figure S6C,G, Table 4 and Table S3), while difference between the R_H_ values of the unmodified PARPs 1 and 2 was significantly less (1.6-fold). These results provide further evidence of the higher effectiveness of PARP1-catalysed PAR chain elongation. The R_H_ value determined for the modified PARP2 after PARG treatment was only slightly higher than the R_H_ value for the unmodified protein (Table 4). Apparently, mono(ADP-ribose) modifications of PARP2 do not essentially increase the R_H_ value of the protein and have therefore no influence on its oligomerization, as it was shown for PARP1 (Table S3).

DLS measurements of the PARP2 hydrodynamic radius upon the PARylation reaction were performed in the presence of various protein partners at the equimolar concentration (Figure S7A). The R_H_ values of associates formed by PARylated PARP2 were determined to be comparable in the absence and presence of either Polβ or APE1 (Table 4). The R_H_ values measured after addition of EDTA (i.e., after disruption of Mg^2+^-coordination bonds) exceeded to a similar extent the respective values measured for the two protein mixtures before the initiation of PAR synthesis (Table 4). Combined, the data suggest that the final level of PARP2 automodification in the reaction conditions used (very high concentrations of PARP2 and NAD^+^) was not modulated by the small protein partners Polβ and APE1. The volume-weighted size distribution profiles observed for the PARP2 binary mixtures with XRCC1 and PARP1 upon the PARylation reaction were presented in each case by two peaks (Figure S9). The R_H_ value determined at the plateau of PARylation reaction for main species of the PARP2 mixture with XRCC1/PARP1 was only 1.5/1.9-fold higher than the respective value before the PARylation reaction (Table 4). Peaks of huge associates presented in the volume-weighted size distribution profiles with R_H_ value of 550 nm for the PARP2-XRCC1 and 750 nm for the PARP2–PARP1 mixture were not visualized in the number-weighted size distribution profiles (Figure S9), indicating small amount of these species in the population. Evidently, the presence of XRCC1 inhibits the elongation step of the PARP2-catalysed reaction, resulting in the synthesis of short PAR chains and thus preventing the formation of intermolecular Mg^2+^-coordination bonds. The R_H_ values determined after addition of EDTA and PARG-catalysed PAR degradation exceeded the respective values for PARP2 alone (6.7 vs. 5.6 and 5.8 vs. 5.2), but were substantially lower than the R_H_ value determined for the PARP2–XRCC1 mixture before the PARylation reaction (7.6). Moreover, the R_H_ value determined after the PAR degradation was lower than the theoretical value for the PARP2–XRCC1 heterotetramer (5.8 vs. 6.44). Taken together, these results suggest that modification of PARP2 even with mono-ADP-ribose influences its interaction with XRCC1, modulating their oligomerization mode. The R_H_ value measured upon the PARylation reaction for the PARP2–PARP1 mixture (14.8 nm) was significantly lower than the respective values for the automodified PARP2 and PARP1 present alone (by 26-fold and 68-fold respectively). These data indicate mutual effects of PARPs on their PARylation and intermolecular association. The R_H_ value determined after disruption of Mg^2+^-coordination bonds was 2.2-fold lower than the respective value for PARP1 alone, indicating strong inhibitory action of PARP2 on elongation and branching of PAR synthesized by PARP1.

### 2.4. Visualization of PARylated Protein Associates Formed by PARP1 and PARP2

**Figure 4:**
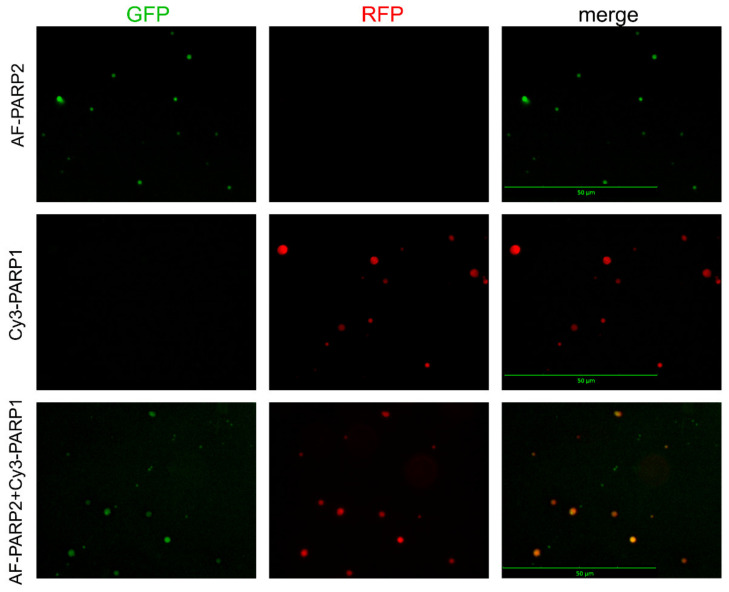
Fluorescence detection by the Celena S Digital Imaging System of protein associates formed upon PARylation reaction catalysed by PARP1, PARP2, and their equimolar mixture. The proteins labelled with distinct fluorophores (AF-PARP2, Cy3-PARP1) were visualized in images acquired with appropriate filters (GFP for AF and RFP for Cy3); the overlapped signals for distinct fluorophores were visualized in the superimposed image (merge). Scale bar, 50 μM. Preparation of samples is detailed in Materials and methods.

For visualization of the associates detected by DLS for PARylated PARP1/PARP2 and their mixtures with each other or BER protein-binding partners, we attempted to apply multicolour fluorescence detection by Celena S Digital Imaging System. Proteins labelled with distinct fluorophores were used to visualize them in protein mixtures. Images acquired with appropriate (specified in Figure legends) fluorescence filters (GFP, RFP, and Cy5) were captured and superimposed to obtain a multifluorescent (merged) image. Images demonstrating presence of fluorescence intensity signals were obtained for protein associates formed upon PARylation reaction catalysed by PARP1 (combined with Cy3-PARP1) or PARP2 (combined with AF-PARP2), using an RFP filter for Cy3-PARP1 and a GFP filter for AF-PARP2 (Figure 4); no signal was visualized in images acquired with the GFP filter for Cy3-PARP1 and RFP filter for AF-PARP2.

Further incubation of samples after the automodification reaction with EDTA excess resulted in disruption of Mg^2+^-coordination bonds [24] and disappearance of fluorescence intensity signals in the images (Figure S10). Thus, the detectable spots represent intermolecular associates formed via coordination of Mg^2^^+^ ions with PAR chains. Fluorescence signals for protein associates formed upon the PARylation reaction catalysed by the equimolar mixture of Cy3-PARP1 with AF-PARP2 were detected in images acquired with both RFP (for Cy3) and GFP (for AF) filters (Figure 4). In the merged image, all spots from the RFP-acquired image (red coloured) overlapped with spots from the GFP-acquired image (green coloured), and only very small green spots specific for AF-PARP2 were visible. The substantial degree of colocalization of PARP1 and PARP2 suggests that the associates formed by the two enzymes present together result from predominant heteroPARylation. The ability of PARP1 and PARP2 to PARylate each other in vitro was shown earlier [13].

**Figure 5:**
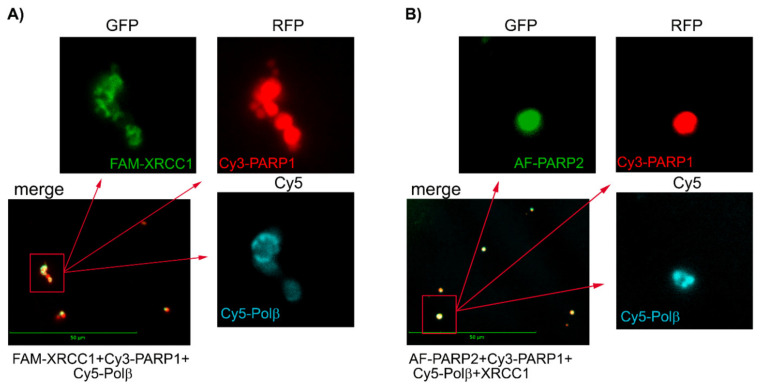
Fluorescence detection of associates formed upon the PARylation reaction catalysed by PARP1 (**A**) or its equimolar mixture with PARP2 (**B**) in the presence of XRCC1 and Polβ. Merged images show colocalization of proteins labelled with distinct fluorophores (as specified in the legend below the image). Enlarged images of selected merged areas in each panel were acquired with filters indicated (above the images).

We performed the fluorescence detection experiments for associates formed by PARP1 and PARP2 in the presence of other BER proteins, using various combinations of proteins labelled with distinct fluorophores. In images acquired for the mixture of Cy3-PARP1 with FAM-XRCC1 and Cy5-Polβ, and the mixture of AF-PARP2 with Cy5-XRCC1 and Cy3-Polβ, both XRCC1 and Polβ were visualized and their fluorescence intensity signals overlapped with the respective signals of PARPs (Figure 5A and Figure S11A). None of the proteins was visualized when the samples were treated with EDTA (Figure S10), indicating that all the spots detected represent intermolecular associates stabilized by Mg^2+^-coordination bonds with PAR chains. Notably, the fluorescence intensity signal of FAM-Polβ detected for its ternary mixture with Cy3-PARP1 and unlabelled XRCC1 was invisible in images for the binary mixture with Cy3-PARP1 (Figure S11B), indicating that colocalization of Polβ with PARylated PARP1 was controlled by the interaction of Polβ with XRCC1. Images acquired for the mixture of AF-PARP2 with Cy3-PARP1, Cy5-Polβ, and XRCC1 (Figure 5B and Figure S11A) show overlapping of the fluorescence intensity signals specific for all three labelled proteins, indicating colocalization of the Polβ-XRCC1 complex with the associates formed by heteroPARylated PARPs. Thus, protein associates detected by DLS to be formed upon PARylation reactions catalysed by PARP1 and PARP2 can be visualized and analysed for their composition, using the multicolour fluorescence detection systems.

## 3. Discussion

Base excision repair (BER) is a multistep DNA repair process in mammalian cells for the processing of most common DNA lesions arising upon oxidative stress and ionizing radiation. The efficiency of BER depends on the coordinated action of enzymes that catalyse the sequential steps [2,3]. One of the coordination mechanisms of BER is the formation of multiprotein complexes (repairosome) composed of enzymes, regulatory and scaffold proteins. PARP1 is one of the key components of repairosome due to its PARylation activity in response to DNA damage and interaction with repair enzymes. PARP1 plays a major role in recruitment to the damaged DNA of XRCC1, a crucial scaffold protein that interacts with multiple BER enzymes and factors [2,31] and has the highest affinity for PAR [32]. The requirement for PARP2 in BER demonstrated in vivo has been proposed to be mediated by its interaction with PARP1 [13]. Despite the accumulated numerous data on PARP2 interaction with BER DNA intermediates and functions of its structural domains [8,15,17,18,19,21,22,33], the exact role of PARP2 in BER remains obscure. PARP2 differs substantially from PARP1 in the modular architecture composed of an N-terminal region (NTR), a central WGR domain, and a C-terminal catalytic (CAT) domain [8]. The NTR is deprived of specific PARP1 three zinc fingers and the BRCT domain required for interaction with DNA and proteins, respectively [2,15]. The WGR domain of PARP2, responsible for the homo- and hetero-oligomerization with PARP1, XRCC1, Polβ, and LigIIIα, has been proposed to combine the functions of the WGR and BRCT domains of PARP1 [13]. All three domains of PARP2 contribute to interaction with damaged DNA [15]. Previously, we characterized complexes of PARP1 formed in solution under true equilibrium conditions with functionally different BER proteins, using quantitative fluorescence-based approaches [23]. The interaction of PARP2 with BER proteins has not yet been quantified. The primary aim of this study was to compare the relative stabilities of complexes formed by PARP2 and PARP1 with common BER protein partners and with each other as well as of their homo-oligomerized complexes for further elucidation of the roles of PARPs in repairosome formation. Apparent equilibrium dissociation constants (EC_50_) of PARP2 complexes with APE1, Polβ, and XRCC1 determined in experiments with FAM-labelled proteins (Table 1) exceed by 1.4−1.9-fold the respective characteristics for the PARP1 complexes [23]. The lower affinity of PARP2 for the proteins results most likely from the absence of the BRCT domain identified as the primary protein binding domain in PARP1 [13,15]. On the other hand, despite the structural differences between PARP2 and PARP1, they both bind APE1 with the highest affinity and both form less stable complexes with Polβ and XRCC1, characterized by comparable dissociation constants. Thus, PARP1 and PARP2 display similar selectivity towards the key BER proteins, suggesting their overlapping functions in coordination of the BER process via direct protein–protein interactions. Very similar EC_50_ values determined for homo-oligomers of PARP1 (130 nM, [23]) and PARP2 (152 nM), and for their hetero-associated complex (146 nM), reinforce the hypothesis that the major in vivo form of the PARP2 protein (less abundant than PARP1) is its hetero-oligomeric complex with PARP1 [13]. The absence of great differences between PARP2 and PARP1 in their binding affinities for the protein partners may result from participation of the conserved CAT domain in the protein–protein interactions. In the dimeric structure of full-length PARP1 determined recently, the interface between two subunits is formed by WGR, BRCT, and CAT domains [34], suggesting that all three domains can be potentially involved in the interaction with protein partners.

The affinity of PARP2 for various protein partners, Polβ, XRCC1, and PARP1, was revealed to be increased in the presence of model DNA duplexes containing SSB (gap-DNA or nick-DNA) (Table 2). This contrasts with our results obtained for PARP1: DNA-induced effects on the affinity of PARP1 for Polβ and XRCC1 were insignificant [23]. It is conceivable that the ternary complexes are stabilized by direct protein–protein and DNA-mediated interactions whose relative contribution is specific for each complex. The DNA-mediated contacts may contribute more significantly to the overall stability of the ternary complexes formed by PARP2 due to unique features. The CAT domain of PARP2 alone has no DNA-binding activity but contributes jointly with the NTR and WGR domains to the affinity of the full-length protein for DNA, indicating that the interdomain contacts mediate interaction with DNA; the DNA-binding NTR domain important for PARP2 activation on SSB participates also in protein–protein interactions [15]. The strongest interaction between PARP2 and PARP1 in the complex with SSB-containing DNA may have biological significance: PARPs 1 and 2 known to fulfil many overlapping functions in SSB repair [9] may cooperate with each other in this process.

To determine the oligomeric states of PARP2 and particularly of its complexes with other BER proteins (never characterized before), we used the DLS technique. In our previous DLS study [24], we proposed that PARP1 in solution exists mainly as a dimer and its complexes with key BER proteins (APE1, Polβ and XRCC1) are heterotetramers (dimers of heterodimers). Dimerization of full-length PARP1 shown by various biochemical approaches was confirmed recently in an electron microscope study [34,35]. DLS experiments of the present study revealed similarity between PARP2 and PARP1 in the oligomeric states of both homo- and hetero-oligomerized complexes. The only exception is the PARP2 complex with APE1 proposed to exist as an equilibrium mixture of heterodimeric and heterotetrameric forms.

Using the DLS technique, we succeeded recently in detection of huge associates formed by PARylated PARP1, that are stabilized via intermolecular Mg^2+^-coordination bonds with elongated and branched PAR polymers [24]. High concentrations of PARP1 and NAD^+^ used in these experiments fall within the range of intracellular concentrations of the enzyme and substrate [36], suggesting the existence of such associates in vivo. Our data provide the first evidence that intracellular compartmentalization through liquid demixing shown for isolated PAR chains [37] can be promoted by the automodified PARP1 itself. It has been further shown that compartmentalization operating by automodified PARP1 and FUS results in the selection of damaged DNA from bulk DNA [38,39]. In the present study, PARP2 was shown to form the intermolecular associates of a smaller size as compared to those for PARP1 due to the lower PARP2 activity in elongation of PAR chain. The higher effectiveness of PARP1-catalysed PAR chain elongation was shown by analysis of the PAR chain length. In addition, DLS measurements performed for the unmodified PARPs 1 and 2 and their PARylated forms in the presence of EDTA (i.e., in conditions preventing formation of the intermolecular associates; Table 4 and Table S3) show that the increment in the R_H_ value of PARP1 due to PARylation is significantly higher as compared to that for PARP2 (13.6 nm vs. 0.6 nm). Thus, for the reaction conditions used, PARP2 catalyses much less efficiently the elongation of PAR polymers than PARP1 does. These results may explain the predominant contribution of PARP1 to overall PAR synthesis in response to DNA damage in vivo [16]. In vitro studies have shown a higher catalytic activity of PARP1 vs. PARP2 in the presence of various activating DNAs [17,22]. In contrast to our data, no significant difference between PARPs 1 and 2 in the length distribution of the PAR polymers has been detected in previous experiments performed at very low enzyme concentration (in a nanomolar range, as compared to the micromolar range in our study) [22]. Taken together, these data suggest that inhibition of the elongation step of PAR synthesis at high PARP concentrations [40] is stronger for PARP2 than for PARP1.

The DLS analysis of PARP2 PARylated in the presence of BER protein partners revealed strong inhibition of the automodification reaction by XRCC1 as shown by the 34-fold decrease in the R_H_ value of the intermolecular associate. In our previous DLS experiments performed for PARP1 under the same reaction conditions, no effect of XRCC1 on the size of PARylated PARP1 was detected. XRCC1 has been shown to regulate activity of both PARP1 and PARP2, assayed at a limited NAD^+^ concentration, but with different efficiency: the activity of PARP2 was significantly inhibited by the equimolar XRCC1 concentration, while at least a 4-fold XRCC1 excess was required to suppress the PARP1 activity [13,41]. Our results provide evidence that the activity of PARP2 even at a high (close to the physiological level) concentration of the NAD^+^ substrate is more effectively regulated by XRCC1.

DLS measurements of PARylated species enabled us to reveal that the intermolecular associates formed by the equimolar PARP2–PARP1 mixture were much smaller in size than those detected for PARP2 and PARP1 present alone, and PARylated species generated after disruption of Mg^2+^-coordination bonds were smaller than the respective species detected for PARP1 alone (Table 4 and Table S3), indicating strong inhibition of PAR chain elongation. The ability of PARP2 to suppress activity of PARP1 shown in previous studies was explained by competition between the PARPs for the DNA binding and alternative formation of a less active hetero-oligomerized complex [17,22]. The hetero-oligomerization of PARP1 and PARP2 was favoured in our reaction conditions: the concentration of PARPs significantly exceeded the apparent dissociation constant of the complex. Moreover, we showed that the complex of PARP2 with PARP1 was further stabilized by the interaction with SSB-containing DNA. Thus, our study provides the first evidence that PARP2 can regulate the activity of PARP1 via hetero-oligomerization of the proteins in complex with damaged DNA. Using the fluorescence Imaging System, we visualized intermolecular associates formed in vitro by the autoPARylated and heteroPARylated PARP1 and PARP2 and revealed colocalization of XRCC1 and Polβ with different size associates (formed by PARP1 in the absence or presence of PARP2). Both XRCC1 and Polβ are PAR-binding proteins, but XRCC1 has significantly higher affinity [32,42]. Invisibility of Polβ in the absence of XRCC1 indicates that their colocalization with PARylated associates is controlled by strong interaction of XRCC1 with Polβ [23] and PAR.

**Figure 6:**
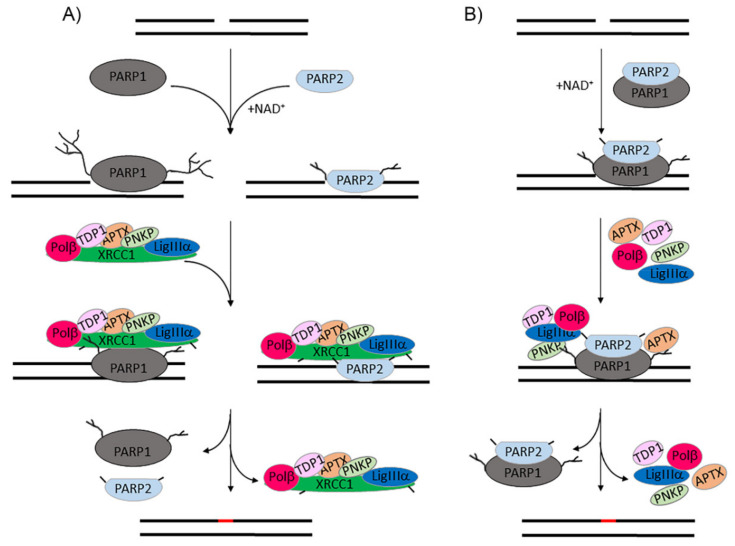
Functions of PARP2 in BER. (**A**) PARP1 and PARP2 perform overlapping functions in SSB detection with following activation and autoPARylation, and PAR-mediated recruitment of XRCC1 and BER enzymes involved in processing DNA damage. The activities of PARPs 1 and 2 are unequally regulated by XRCC1; the more efficient inhibition of PARP2 suggests its prevailing contribution at limited XRCC1 concentrations. (**B**) Hetero-oligomerization of PARP2 with PARP1 negatively regulates the efficiency of PAR synthesis. The autoregulatory behaviour of the PARP1–PARP2 complex enables SSB repair through an XRCC1-independent subpathway.

Numerous studies have highlighted redundancy between PARP1 and PARP2 in BER due to their overlapping functions in SSB detection and recruitment of BER enzymes and the XRCC1 scaffold protein via direct and PAR-mediated protein–protein interactions [6,9,10]. These results are extended in our study, providing the first quantitative evidence for the similarities and differences between PARPs 1 and 2 in the interaction with common BER protein partners. We have shown that the catalytic activity of PARP2 is sufficient to produce the intermolecular associates upon the automodification reaction, which may promote intracellular compartmentalization and thus facilitate assembling the repairosomes in response to DNA damage. Functional significance of PARP1–PARP2 hetero-oligomerization has been clarified: this complex, stabilized specifically by SSB-containing DNA, is responsible for negative regulation of the PARP1 and PARP2 activities, which in turn controls the retention of PARylated PARPs on damaged DNA. The regulatory function of PARP2 in limiting PARP PARylation is similar to that of XRCC1 and might contribute to XRCC1-independent DNA repair. The XRCC1-independent, but Polβ-dependent, mechanism has been shown to exist in vivo for protection against DNA damage [43], but its nature has not yet been elucidated [3]. Taken together, our findings suggest that PARP2 can function differently in BER, depending on relative concentrations of PARP1, PARP2, and XRCC1. PARP2 can replace the traditional function of PARP1, promoting assembling of the repair complex via PAR-mediated recruitment of XRCC1 (Figure 6). The alternative function of PARP1 and PARP2 in the hetero-oligomeric complex, which is capable of autoregulation of the PARylation level, can promote DNA repair without participation of XRCC1. It is likely that hetero-oligomerization of PARP1 with specific protein partners is a key mechanism underlying contribution of PARP1 to DNA repair as well as other cellular processes. The characteristic feature of this mechanism is negative regulation of PAR chain elongation in the automodified PARP1 via its interaction with the protein partner. Such a role of a protein partner assigned for the first time for XRCC1 [41] was further demonstrated for RPA [44] and YB1 [45].

## 4. Materials and Methods

### 4.1. Protein Expression and Purification

Vectors for expression of human poly(ADP-ribose) polymerase 1 (PARP1), murine poly(ADP-ribose) polymerase 2 (PARP2), and bovine poly(ADP-ribose) glycohydrolase (PARG) were kindly provided by Dr. V. Schreiber (Strasbourg University, Strasbour, France); human apurinic/apyrimidinic endonuclease 1 (APE1) and rat DNA polymerase β (Polβ), by Dr. S. H. Wilson (National Institute of Health, Noth Carolina, USA); human X-ray repair cross-complementing protein 1 (XRCC1), by Dr. J. P. Radicella (UMR217 CNRS/CEA, Fontenay aux Roses, France). PARP1 and PARP2 recombinant proteins were expressed in insect cells and purified as described previously [46], with some modifications. Recombinant proteins XRCC1, APE1, and Polβ were expressed in *E. coli* and purified according to refs. [47,48,49]. Homogeneity of the purified proteins was verified by SDS-PAG electrophoresis. The XRCC1 protein was contaminated with a small amount (~5%) of its proteolytic fragments (as proved by immunoblotting); the other proteins under study were homogeneous. The enzymatic activities of Polβ, APE1, PARP1, and PARP2 were verified as described previously [17,23]. The PARG protein was expressed in *E. coli*, purified, and kindly provided by E. Ilina (ICBFM SB RAS, Novosibirsk, Russia). The purified proteins were dialyzed against a solution containing 50 mM Tris-HCl, pH 8.0, 200 mM NaCl, 5 mM DTT, and 40% glycerol and stored at −30 °C.

### 4.2. Oligonucleotide Substrates

DNA oligonucleotides were synthesized and purified in the Laboratory of Biomedicinal Chemistry (ICBFM SB RAS, Novosibirsk, Russia). Sequences of oligonucleotides were as follows: template–5′-GGAAGACCCTGACGTTACCCAACTTAATCGCC-3′, complementary oligonucleotide–5′-GGCGATTAAGTTGGGTAACGTCAGGGTCTTCC-3′, upstream primer 1–5′-GGCGATTAAGTTGGG-3′ and primer 2–5′-GGCGATTAAGTTGGGT-3′, downstream primer–5′-pAACGTCAGGGTCTTCC-3′. Double-stranded oligonucleotides (32-mer) containing a one-nucleotide gap (gap-DNA) or nick (nick-DNA) were prepared by annealing the upstream primer 1 or 2, respectively, and the downstream primer to the template oligonucleotide. A non-gapped 32-mer DNA (ds-DNA) was prepared by annealing the complementary oligonucleotide to the template. In each case, an equimolar mixture of the components was heated at 90 °C for 2 min and then slowly cooled down to room temperature.

### 4.3. Dynamic Light-Scattering (DLS) Studies of PARP2 and Its Protein Complexes, and PARP2-Catalysed Poly(ADP-ribosyl)ation

DLS measurements were performed using a Zetasizer Nano ZS (Malvern Instruments Ltd., Malvern, UK) according to the method described in our previous work [24]. Briefly, samples of individual proteins (PARP2, APE1, Polβ, XRCC1, and PARP1) at final concentrations of 6 μM or equimolar mixtures of proteins were prepared in a DLS buffer consisting of 25 mM HEPES-NaOH, pH 7.5, 100 mM NaCl, and 1 mM DTT. All stock solutions and proteins were ultrafiltered by using 0.2 μm pore size PES membrane vivaspin centrifugal concentrator (Sartorius). The measurements and data processing were performed as described previously.

Poly(ADP-ribosyl)ation (PARylation) reaction was carried out directly in a ZEN 2112 low-volume quartz cuvette used for DLS measurements. The reaction mixture (20 μL) contained 25 mM HEPES-NaOH, pH 7.5, 100 mM NaCl, 1 mM DTT, 10 mM MgCl_2_, 6 μM gap-DNA, 6 μM PARP2. Mixtures were equilibrated for 60 s prior to initiation of reaction by NAD^+^ addition to a 2.5 mM concentration. When indicated, samples contained 6 μM protein (APE1, Polβ, XRCC1 or PARP1). After 15-min incubation, the reaction was stopped by the addition of EDTA to a 10 mM concentration. The R_H_ value measurement was performed every 3 min during the reaction and immediately after EDTA addition. Then, the mixture was subjected to further treatment with 0.25 μM PARG for 18 h at 4 °C, and the R_H_ value of the PARylated protein after enzymatic degradation was measured.

### 4.4. Fluorescent Labelling of APE1, Polβ, PARP1, XRCC1, and PARP2

The N-succinimidyl esters (SE) of 5,6-carboxyfluorescein (5,6-FAM-SE; Sigma-Aldrich), 5-Alexa Fluor 488 (AF488-SE; Lumiprobe), sulfo-Cyanine 3 (Cy3-SE; Lumiprobe) or sulfo-Cyanine 5 (Cy5-SE; Lumiprobe) were dissolved in dimethylsulfoxide up to 10 mM concentration and used for protein labelling. The reaction mixture contained 100 mM MES, pH 7.0, 150 mM NaCl, 80−100 μM protein (pre-dialyzed against the reaction buffer), and 1.6−3-fold molar excess of the reagent over the protein. The reaction was carried out for 18 h at 4 °C in the dark and stopped by the addition of ethanolamine up to 2-fold excess over the reagent. Then, labelled proteins (except Cy3-PARP1) were dialyzed exhaustively against buffer A containing 100 mM HEPES, pH 8.0, 200 mM NaCl, and 10 mM DTT, to remove the free dye. Cy3-labelled PARP1 protein was purified from free dye by two successive chromatographies on HiTrap Capto S and Mono S columns (GE Healthcare) equilibrated with buffer B (50 mM Tris-HCl, pH 8.0, 100 mM NaCl, 10 mM β-ME, 0.5 mM EDTA, 5% (*v*/*v*) glycerol) using a 0.1−1.0 M gradient of NaCl concentration in buffer B. The labelled proteins were stored at −30 °C in a solution containing 50 mM HEPES, pH 8.0, 100 mM NaCl, 5 mM DTT, and 40% glycerol. The extent of protein labelling was quantified spectrophotometrically with a CLARIOstar microplate spectrometer (GMB Labtech GmbH, Ortenberg, Germany), using the absorption coefficient of the protein based on the Expasy Protparam Data and the absorption coefficients for dyes taken from Lumiprobe protocols [26]. The purity of labelled proteins was verified by SDS-PAG electrophoresis. The enzymatic activities of PARP1 and PARP2 in autoPARylation reaction were tested using ^32^P-labelled NAD^+^ as described in Supplementary Methods. The activities of Polβ in DNA synthesis and of APE1 in endonucleolytic DNA cleavage were verified as detailed previously [23].

### 4.5. Fluorescence Studies of PARP2 Interaction with Proteins and DNAs

Fluorescence intensities of labelled proteins (used at the fixed concentration of 40 nM) were measured in the absence and presence of various concentrations of the potential partner (protein or DNA). In some cases, the experiments were performed in the presence of the second protein or DNA partner pre-added at sub-saturating concentration (specified in Figure legends). The binding buffer contained 50 mM Hepes (pH 8.0), 100 mM NaCl, and 5 mM DTT. The measurements were performed in low-volume nontransparent polypropylene plates (Corning, NY, USA) using a microplate CLARIOstar spectrometer. The fluorescence intensity for 5,6-FAM and AF488 fluorophores was detected at the excitation wavelength of 482 nm and the emission wavelength of 530 nm. The excitation and emission wavelengths of 530 nm and 580 nm, respectively, were used for fluorescence intensity detection of Cy3-labelled proteins. In each independent experiment, fluorescence intensity was measured at each concentration of the protein/DNA partner in three repeats. The experimental data were processed quantitatively with the MARS Data analysis program (GMB Labtech GmbH, Ortenberg, Germany). The binding curves were described by the equation:

F = F_0_ + (F_∞_ − F_0_)/[1 + (EC_50_/C)^n^] (1)

where F_0_, F, and F_∞_ are fluorescence intensities of the labelled protein in the absence of other proteins, in the presence of protein partner at the given (C) and saturating concentrations, respectively; EC_50_ is the concentration of protein partner at which F − F_0_ = (F_∞_ − F_0_)/2; n is the Hill coefficient. All experiments were performed at least three times.

To detect protein–protein interactions by the FRET approach, the fluorescence intensity of the AF-labelled PARP2 (donor-labelled probe) was measured in the absence and presence of various concentrations of the Cy3-labelled PARP1 (acceptor probe) as we detailed in our previous work [23]. FRET efficiency (E) was calculated from the fractional decrease of the donor fluorescence (F_d_) due to the presence of the acceptor (F_da_):

E = 1 − F_da_/F_d_. (2)

In studying the effects of model DNA substrates on the PARP2 interaction with PARP1, XRCC1 or Polβ, the AF-PARP2/FAM-PARP2 (40 nM) was premixed with the respective DNA at an excessive concentration (400 nM), and the fluorescence intensity observed was taken as a starting F_0_ value.

### 4.6. Visualization of PARP1 and PARP2 Associates Formed upon PARylation Reaction

The PARylation reaction catalysed by PARP1/PARP2 was performed in a mixture (10 μl) containing 2 mM NAD^+^, 1 μM gap-DNA, 50 mM Tris-HCl buffer (pH 8.0), 100 mM NaCl, 10 mM MgCl_2_, 1 μM PARP1 (an equimolar mixture of PARP1 and Cy3-PARP1) or 1 μM PARP2 (an equimolar mixture of PARP2 and AF-PARP2) in the absence or presence of 1 μM labelled protein (AF-PARP2, Cy3-PARP1, FAM-XRCC1, Cy5-XRCC1 or Cy5-Polβ). Mixtures were incubated for 30 min at 37 °C and then placed on ice to terminate the reaction. After addition of PEG 20K to a 0.8% concentration, the reaction mixture aliquot (6 μl) was placed between the microscope slide and 0.17 mm coverslip. Fluorescence images were captured using a Celena S Digital Imaging System with GFP, RFP, Cy5 filter cubes, and Plan Apochromat Fluor Oil 100X (Coverslip-Corrected, NA 1.25, WD 0.19) objective.

## Figures and Tables

**Table 1:** Binding parameters of PARP2 interaction with BER proteins determined by fluorescent titration.

Labelled Protein ^1^	Protein Partner	EC_50_^2^, nM	E ^3^
FAM-PARP2	PARP2	152 ± 12	
FAM-PARP2	Polβ	185 ± 18 *	
FAM-PARP2	XRCC1	190 ± 18 *	
FAM-PARP2	PARP1	146 ± 10	
FAM-APE1	PARP2	61 ± 8 ***	
AF-PARP2	PARP1	79 ± 7 ***	
AF-PARP2	Cy3-PARP1	76 ± 7 ***	0.36 ± 0.04
Cy3-PARP1	PARP2	90 ± 6 ***	
Cy3-XRCC1	PARP2	196 ± 10 **	

^1^ Titration experiments were performed at a constant concentration of the labelled protein (40 nM). ^2^ EC_50_ value derived from the titration curves by fitting to the four-parameter equation is the half-maximal effective concentration of the protein partner, at which F − F_0_ = (F_∞_ − F_0_)/2. Values are the mean (± SD) of 3–5 independent experiments. Values for PARP2–protein complexes, which are statistically different from that for the FAM-PARP2–PARP1 complex (underlined): *p* < 0.05 (*), *p* < 0.01 (**), *p* < 0.001 (***); *t*-test. ^3^ Efficiency of FRET calculated from the experimentally determined fractional decrease of the fluorescence intensity: E = 1 − F_da_/F_d_, where F_da_ and F_d_ are fluorescence intensities of the AF-labelled PARP2 (donor) measured in the presence of Cy3-labelled (acceptor) or unlabelled PARP1, respectively. Relative intensity of fluorescence excited at 482 nm was monitored at 530 nm. F_da_ values were corrected for the contribution of the Cy3-labelled PARP1 (measured by titration of the unlabelled PARP2 with the Cy3-labelled PARP1). Values are the mean (± SD) of three independent experiments.

**Table 2:** Effects of model DNAs on the PARP2 interaction with proteins.

Labelled Protein ^1^	DNA ^2^	Protein Partner	EC_50_^3^, nM	Effect on Binding Affinity ^4^	Effect on FRET Efficiency ^5^
FAM-PARP2	gap-DNA	PARP2	101 ± 9 **	1.5 (+)	
FAM-PARP2	gap-DNA	Polβ	106 ± 10 **	1.7 (+)	
FAM-PARP2	gap-DNA	XRCC1	164 ± 12 *	1.2 (+)	
AF-PARP2	nick-DNA	PARP1	44 ± 5 **	1.8 (+)	
AF-PARP2	ds-DNA	PARP1	144 ± 16 **	1.8 (−)	
AF-PARP2	nick-DNA	Cy3-PARP1			−0.04 *
AF-PARP2	ds-DNA	Cy3-PARP1			+0.08 **
FAM-PARP2	gap-DNA		≤18		
AF-PARP2	nick-DNA		≤15		
AF-PARP2	ds-DNA		35 ± 4		

^1^ Labelled PARP2 protein (40 nM) premixed with DNA (400 nM) was titrated by a protein partner. ^2^ Model DNAs represent BER DNA intermediates: gap-DNA–DNA with a single nucleotide gap, nick-DNA–DNA with a single-strand break and an internal 5′-phosphate, ds-DNA–DNA duplex without damage. ^3^ EC_50_ value is the half-maximal effective concentration of the binding partner, at which F − F_0_ = (F_∞_ − F_0_)/2. Values are the mean (± SD) of 3–5 independent experiments. Values determined for each protein pair in the presence of DNA, which are statistically different from the respective value in the absence of DNA (Table 1), are marked *p* < 0.05 (*), *p* < 0.01 (**). Typical titration curves used to determine EC_50_ values for complexes of FAM/AF-labelled PARP2 with DNAs are presented in Supplementary Materials, Figure S3. ^4^ Degree of increase (+) or decrease (–) in the binding affinity in the presence of DNA. ^5^ Increase (+) or decrease (–) in FRET efficiency between AF- and Cy3-labelled proteins in the presence of DNA. The respective mean E values were determined in independent measurements (*n* = 5–7) at three (four) sub-saturating PARP1 and Cy3-PARP1 concentrations (200–400 nM); statistically significant changes in the E value produced by DNAs are marked *p* < 0.05 (*), *p* < 0.01 (**).

**Table 3:** Hydrodynamic radii and the most probable oligomeric states of PARP2 and its heterocomplexes with BER proteins.

Protein(s) ^1^ (Mw ^2^, kDa)	DLS R_H_ ^3^, nm	Theoretical R_H_ ^4^, nm		Oligomeric State ^5^
		Protomer	Dimer (Tetramer)	
Homo- and hetero-oligomerization of PARP2				
PARP2 (62.0)	5.0 ± 0.4	3.47	4.67 (6.28)	A_2_
PARP2 + Polβ (100.3)	5.6 ± 0.3	4.27	5.74	(AB)_2_
PARP2 + APE1 (97.5)	4.9 ± 0.2	4.21	5.67	AB+(AB)_2_
PARP2 + XRCC1 (131.5)	7.6 ± 0.3	4.79	6.44 (8.66)	(AB)_2_
PARP2 + PARP1 (175.0)	7.8 ± 0.6	5.41	7.28	(AB)_2_
Homo-oligomerization of BER proteins determined in our previous study [24]				
Polβ (38.3)	3.5 ± 0.2	2.83	3.80	A_2_
APE1 (35.5)	3.2 ± 0.2	2.74	3.68	A_2_
XRCC1 (69.5)	5.7 ± 0.2	3.65	4.90	A_2_
PARP1 (113.0)	8.0 ± 0.5	4.50	6.04(8.12)	A_2_ ^6^

^1^ Solutions of individual proteins and their equimolar mixtures at a concentration of 6 μM were analysed by DLS. ^2^ Molecular weight of the protomer (monomer or heterodimer) calculated from the protein sequences of constituent protein(s) (retrieved from the ExPASy Bioinformatics Research Portal). ^3^ Experimentally determined average hydrodynamic radius (R_H_) for species from the volume-weighted size distribution. Values are the mean (± SD) of three independent experiments. ^4^ The theoretical R_H_ value for the protomer and its oligomer (two protomers) was calculated from the molecular weight, assuming the globular shape of proteins and complexes. ^5^ Oligomeric forms are designated: A_2_–homodimer; AB/(AB)_2_–heterodimer/heterotetramer (dimer-of-heterodimers). ^6^ Higher-order oligomerization is possible.

**Table 4:** DLS analysis of PARylated PARP2.

Protein(s) ^1^	R_H_ ^2^, (nm)			
	Before Reaction Initiation	PARylated Protein Associate ^3^	PARylated Protein after Addition of EDTA ^3^	PARylated Protein after PARG Treatment ^3^
PARP2	5.0 ± 0.4	383 ± 45	5.6 ± 0.3	5.2 ± 0.3
PARP2 + Polβ	5.6 ± 0.3	370 ± 43	6.2 ± 0.5	5.8 ± 0.2
PARP2 + APE1	4.9 ± 0.2	440 ± 51	5.2 ± 0.3	n. d. ^4^
PARP2 + XRCC1	7.6 ± 0.3	11.3 ± 1.6	6.7 ± 0.5	5.8 ± 0.4
PARP2 + PARP1	7.8 ± 0.4	14.8 ± 1.4	9.9 ± 1.2	n. d. ^4^

^1^ The concentration of proteins (individual and in the equimolar mixtures) was 6 μM. ^2^ Experimentally determined average R_H_ value of species in single (major) peak of volume-weighted size distribution. Values are the mean (± SD) of three independent experiments. ^3^ Poly(ADP-ribose) synthesis was performed in the presence of Mg^2+^ for 15-min incubation; further treatment with EDTA and PARG was performed as detailed in Materials and methods. ^4^ Not determined.

## Data Availability

Not applicable.

## Supplementary Materials

The following are available online at https://www.mdpi.com/article/10.3390/ijms22094679/s1.

## Abbreviations

PARP1	poly(ADP)ribose polymerase 1
PARP2	poly(ADP)ribose polymerase 2
Polβ	DNA polymerase β
XRCC1	X-ray repair cross-complementing protein 1
APE1	apurinic/apyrimidinic endonuclease 1
BER	base excision repair
SSB	single-strand break
FAM	5(6)-carboxyfluorescein
AF488	5-Alexa Fluor 488
Cy3	sulfo-Cyanine 3
Cy5	sulfo-Cyanine 5
SE	N-succinimidyl ester
FRET	fluorescence resonance energy transfer
DLS	dynamic light scattering
PAR	poly(ADP-ribose)
