# Supplementary material for: Functional Roles of PARP2 in Assembling Protein–Protein Complexes Involved in Base Excision DNA Repair
Source: Int J Mol Sci. 2021 Apr 28;22(9):4679. doi: 10.3390/ijms22094679 (PMC8124814; doi:10.3390/ijms22094679)
Supplement: Supplementary file 1 [file ijms-22-04679-s001.zip › ijms-1174394-sup-FOR XML.pdf]

International Journal of
Molecular Sciences

MDPI

Supplementary material

Functional roles of PARP2 in assembling protein-protein complexes involved in base excision DNA repair

Inna Vasil'eva [1,#], Nina Moor [1,#], Rashid Anarbaev [1], Mikhail Kutuzov [1], Olga Lavrik [1,2,*]

[1] Institute of Chemical Biology and Fundamental Medicine, Siberian Branch of the Russian Academy of Sciences, Novosibirsk, 630090, Russia; iva@niboch.nsc.ru (I.V.); moor@niboch.nsc.ru (N.M.); anarbaev@niboch.nsc.ru (R.A.); kutuzov.mm@mail.ru (M.K.); lavrik@niboch.nsc.ru (O.L.)
[2] Novosibirsk State University, Novosibirsk, 630090, Russia
* Correspondence: lavrik@niboch.nsc.ru (O.L.)
# These authors contributed equally to this paper

Supplementary Methods

1. *Glutaraldehyde cross-linking of proteins*

Cross-linking reaction was performed in DLS buffer containing 25 mM HEPES-NaOH, pH 7.5, 100 mM NaCl and 1 mM DTT. The individual proteins and their equimolar mixtures (3 μM of each) were incubated before addition of glutaraldehyde for 30 min at 4°C. Cross-linking was performed by addition of glutaraldehyde to a final concentration of 0.05% and incubation for 15 min at 25°C. The reaction was quenched by addition of $NaBH_4$ to a concentration of 20 mM and the samples were kept for 30 min at 4°C. SDS-PAGE sample buffer was then added to the samples. The reaction products were analysed by SDS-PAGE using 5% stacking gel (1.25% C) and 10% separating gel (3.33% C). The gel was subjected to visualization and quantifying using Typhoon FLA9500 and Quantity One software, then it was stained with a Coomassie blue and dried. Proteins labelled with 5(6)-carboxyfluorescein (FAM) at the terminal amino group used in the cross-linking experiments were obtained as described previously [1].

2. *Analysis of poly(ADP-ribosyl)ated PARP1/PARP2 by gel electrophoresis*

The automodification reaction catalysed by PARP1/PARP2 was performed in a reaction mixture containing 25 mM HEPES-NaOH, pH 7.5, 100 mM NaCl, 1 mM DTT, 10 mM $MgCl_2$, 2 μM gap-DNA, 2.4 mM NAD^+, and 2 μM [^{32}P]NAD^+. The reaction was initiated by adding PARP1/PARP2 to a final concentration of 6 μM. Samples were incubated at 25 °C, and aliquots were taken at 1, 3, 6, 10 and 15 min. The reaction was terminated by addition of a SDS-PAGE sample buffer and heating for 2 min at 90 °C. The PARG catalysed hydrolysis of PAR in autoPARylated PARP1/PARP2 proteins was performed by addition of 10 mM EDTA and 0.25 μM PARG to the reaction mixture aliquot (after 15-min incubation) with following incubation for 30 min at 37 °C. Products of the PARylation reaction, without and with PARG treatment, were separated by 10% SDS-PAGE and then analysed by phosphorimaging. The chain length of PAR polymer detached from the PARylated proteins by alkaline hydrolysis was analysed as described previously [2].

Supplementary Results

Detection and quantification of PARP2–protein interactions by fluorescence titration

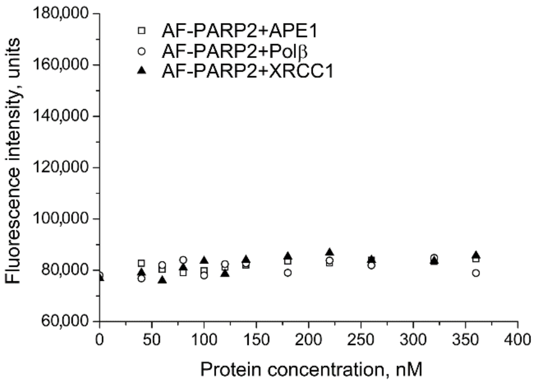

Figure S1. Fluorescence titration of AF-PARP2 with APE1, Polβ and XRCC1. AF-PARP2 (40 nM) was excited at 482 nm in the absence or presence of increasing concentrations of the unlabelled protein partner and the relative fluorescence intensities were monitored at 530 nm. The data demonstrate no detectable change in the fluorescence intensity of AF-PARP2 due its binding to the proteins.

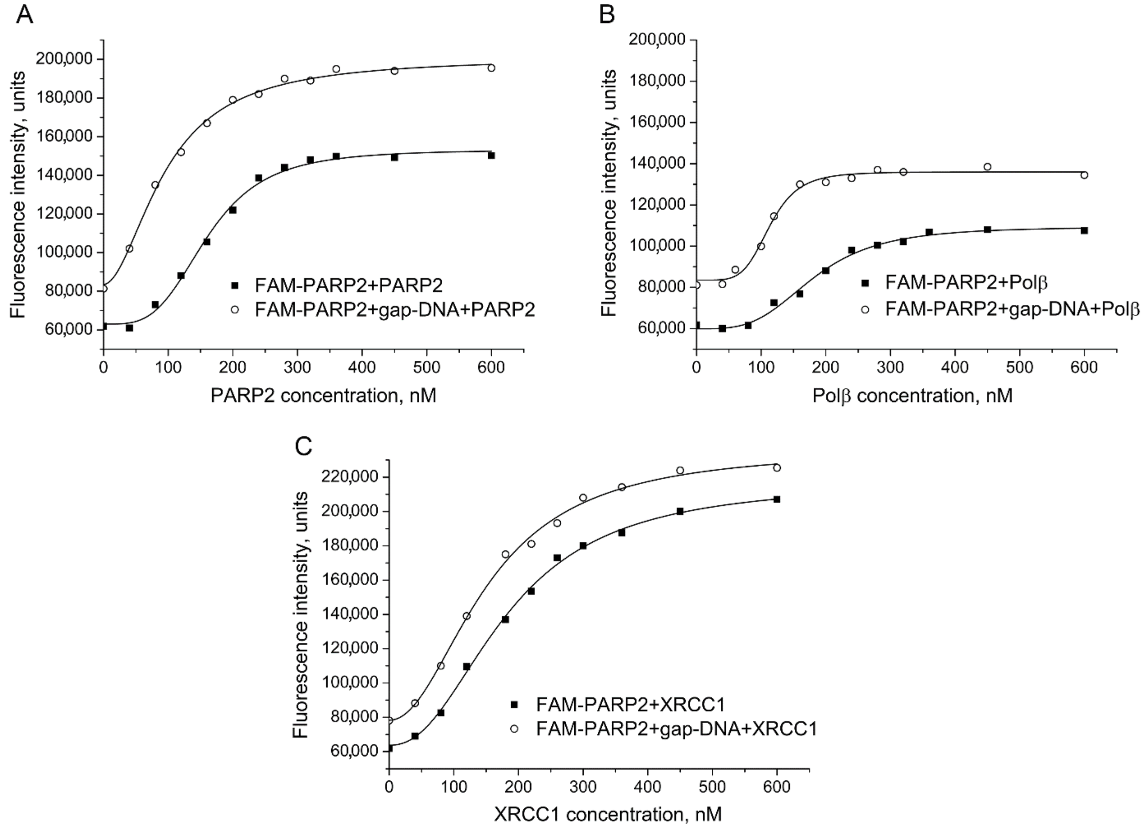

Figure S2. Influence of gap-DNA, a key DNA intermediate of BER, on homo-oligomerization of PARP2 (A) and its interaction with Polβ (B) and XRCC1 (C). Fluorescence titration of FAM-PARP2 with the unlabelled protein partner was performed in the absence (filled symbols) or presence (opened symbols) of gap-DNA (400 nM); higher values of F_0 measured in DNA presence are indicative of protein-DNA binding. Curves show the best fits of the four-parameter equation (with R^2 values exceeding 0.97).

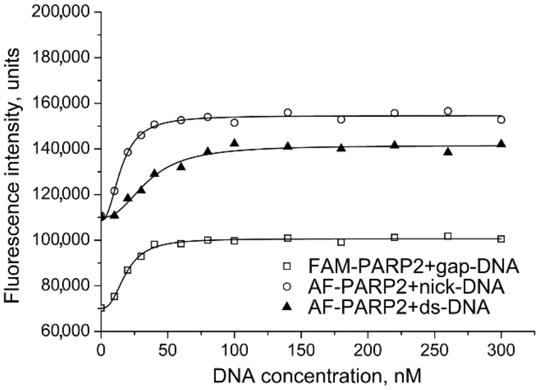

Figure S3. Fluorescence titration of FAM/AF-labelled PARP2 with various model DNAs: gap-DNA, nick-DNA and ds-DNA. Curves show the best fits of the four-parameter equation (with R^2 values exceeding 0.97). The EC_{50} values determined for the PARP2 complexes with gap-DNA (≤ 18 nM) and nick-DNA (≤ 15 nM) are significantly lower than the concentration of the labelled protein (40 nM) and therefore represent the upper limit for the equilibrium dissociation constant of these complexes.

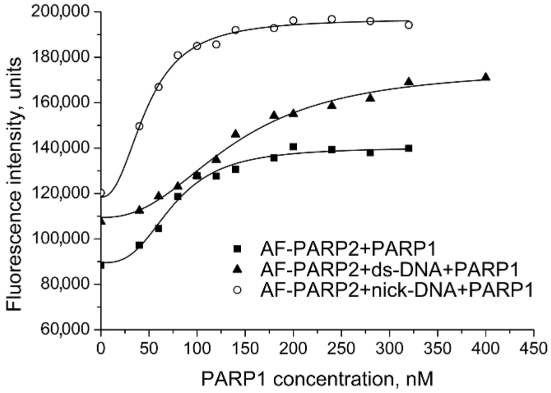

Figure S4. Modulation of the PARP2-PARP1 interaction by model DNAs. Fluorescence titration of AF-PARP2 (40 nM) with the unlabelled PARP1 was performed in the absence or presence of nick-DNA or ds-DNA (400 nM); higher values of F_0 measured in DNA presence are indicative of PARP2-DNA binding. Curves show the best fits of the four-parameter equation (with R^2 values exceeding 0.97).

Cross-linking analysis of proteins homo- and hetero-oligomerization

We performed glutaraldehyde cross-linking experiments to compare homo- and hetero-oligomerization of proteins with various binding partners in binary mixtures. A high reactivity of glutaraldehyde and the nonselective reaction with the amino groups of amino acid residues results in formation of primary cross-linked species, including intramolecular cross-links with similar electrophoretic mobility, and of secondary high molecular weight products that are difficult to resolve completely. Despite these limitations the glutaraldehyde cross-linking approach is useful to compare strength of interaction between proteins and to identify structural domains responsible for the interaction [2]. In preliminary experiments, the glutaraldehyde concentration was optimized to detect all possible cross-linked species formed by small and large proteins used in our study. The results presented were obtained at the glutaraldehyde concentration of 0.05%. To identify and quantify relative yields of cross-linked products formed in the mixtures of various proteins, FAM-labelled Polβ and APE1 were used.

Cross-linking performed for the individual proteins, Polβ, APE1, XRCC1 and PARP2, revealed several species visualized by fluorescence imaging and gel staining (Fig. S5). Products migrated below the band corresponding to the native (untreated) protein were identified as intramolecular cross-links (specified as A on the histograms). Products migrated with an apparent molecular weight close to that of dimer/tetramer were identified as cross-linked homodimers/homotetramers (A_2/A_4). Cross-linked species with the lowest mobility were specified as cross-linked multimers (higher order complexes). Distribution of fluorescence intensity among the cross-linked products of FAM-Polβ and FAM-APE1 present alone (Fig. S5A, lanes 7 and 9, and B) shows a half of the proteins to form the intramolecular cross-links. Among the cross-linked species formed by XRCC1 the intramolecular cross-links were also detected as the major ones (Fig. S5A, lane 11). Approximately half of the cross-linked species formed by PARP2 were intramolecular, and the remaining ones were specified as cross-linked multimers (Fig. S5A, lane 6).

The cross-linking experiments performed for the binary mixtures of FAM-Polβ and FAM-APE1 with PARP2 revealed formation of the intramolecular cross-links and cross-linked homodimers of the FAM-labelled proteins (Fig. S5A, lanes 8 and 10, and B). Higher yields of multimeric cross-links as compared to those for the FAM-labelled proteins cross-linked alone indicate the hetero-oligomerization of Polβ and APE1 with PARP2. The increased yield of multimers detected for the binary PARP2-XRCC1 mixture as compared to the cross-linked individual PARP2 and XRCC1 (Fig. S5A, lane 12 compared to lanes 6 and 11) indicates a higher propensity of the hetero-complex to form the cross-linked aggregates.

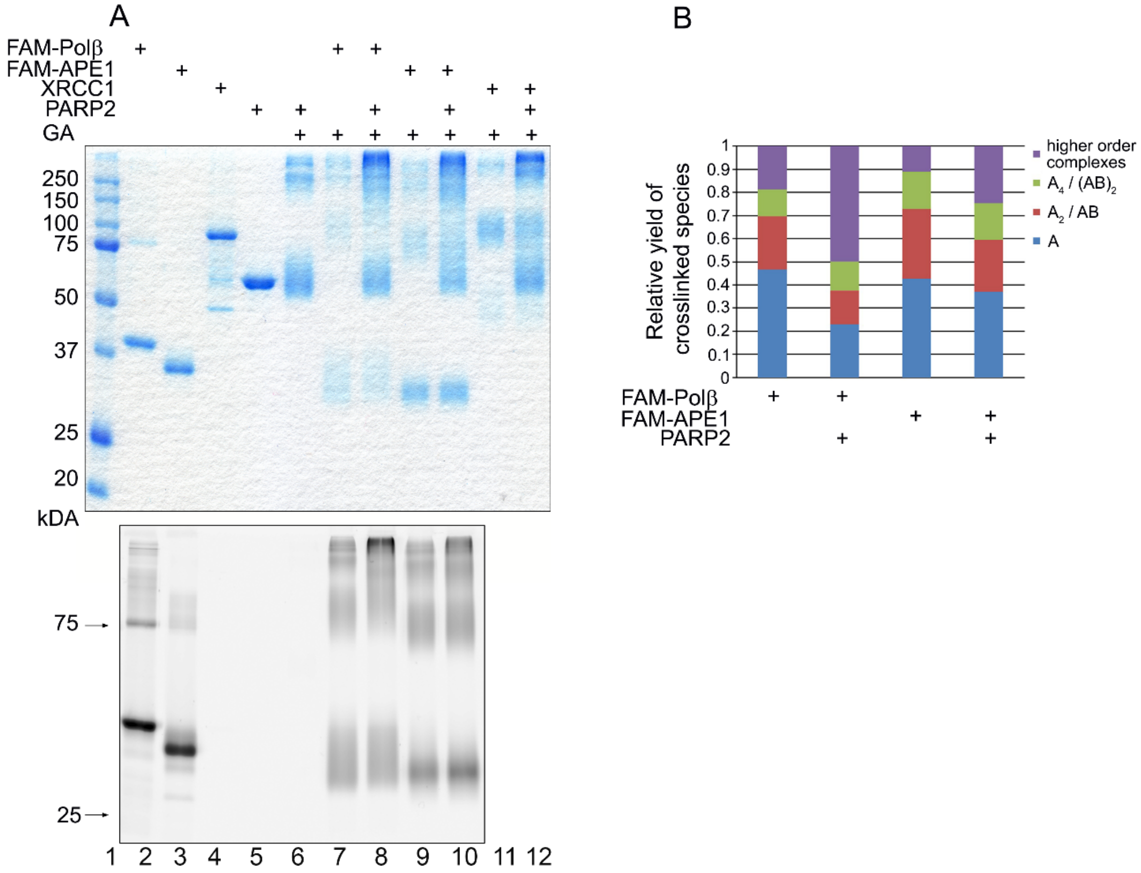

Figure S5. Glutaraldehyde cross-linking of PARP2 in the absence and presence of various BER protein partners. Cross-linking of FAM-Polβ, FAM-APE1, XRCC1 or PARP2 was performed for the proteins present alone or in the equimolar mixture of PARP2 with the other one protein. The reaction conditions are described in Supplementary Methods. (A) The cross-linked products were separated by electrophoresis in 10% SDS-PAG, and visualized by Coomassie blue staining (upper image) and fluorescence imaging (lower image). Histogram (B) shows relative yields of cross-linked species formed by FAM-labelled Polβ and APE1 determined as the relative fluorescence intensity of bands detected in each sample. Designation of homo- and hetero-associated cross-linked products is specified in the text.

DLS study of PARP2 interaction with BER proteins

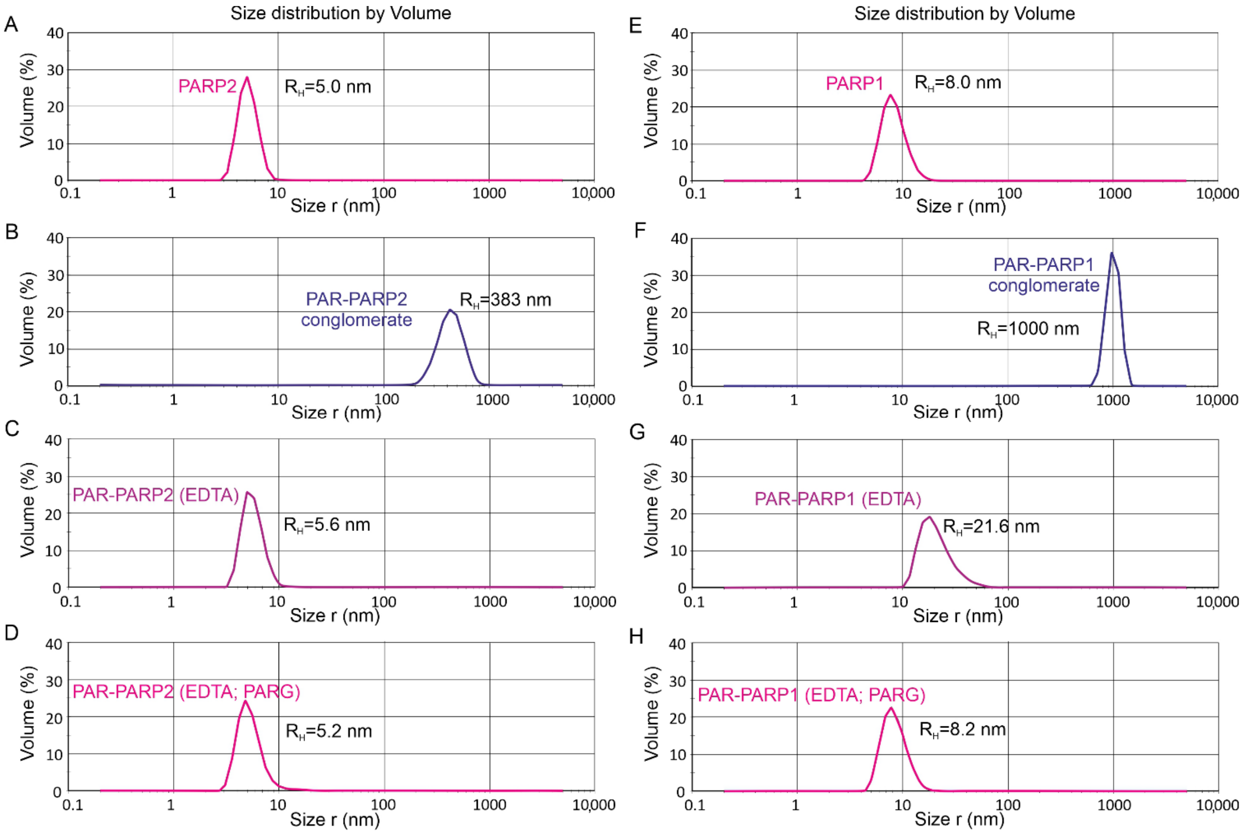

Figure S6. Comparison of the volume-weighted size distribution profiles for PARP2 and PARP1 proteins (A, E) and their PARylated forms: PAR-PARP2 (B-D) and PAR-PARP1 (F-H). The R_H values were measured directly after 15-min incubation with NAD^+ and DNA (when the size growing reached the plateau) (B, F), after the following EDTA addition (C, G) or PARG treatment (D, H).

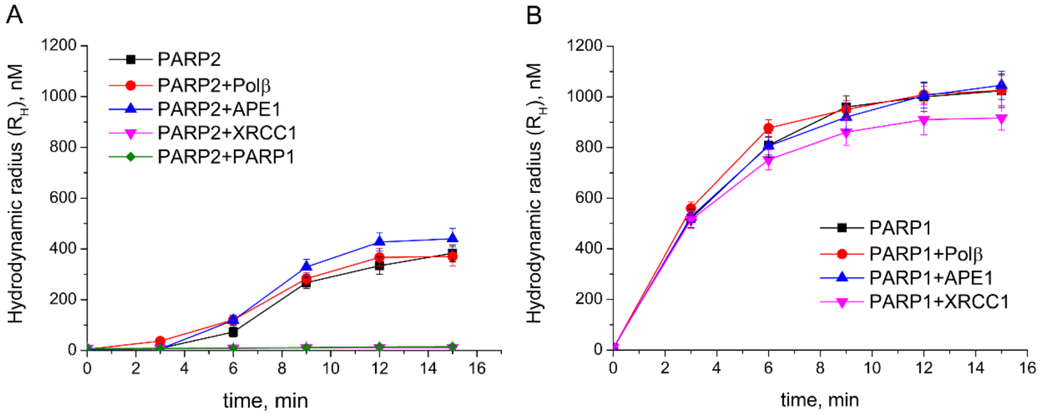

Figure S7. Kinetic measurements of the hydrodynamic radius for PARylated PARP2 (A) and PARP1 (B). The reaction was performed in absence or presence of the interacting protein (Polβ, APE1, XRCC1 and PARP1) at the equimolar concentration.

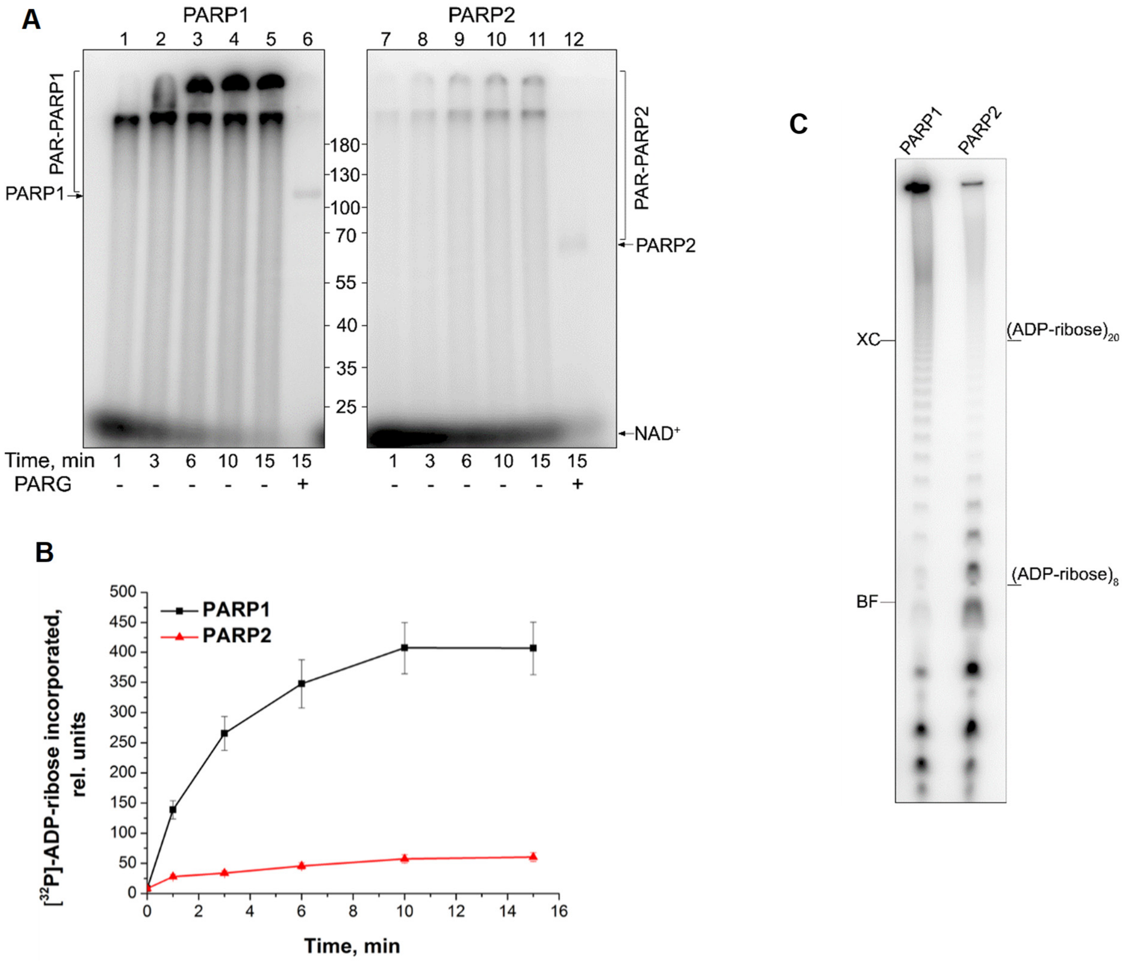

Figure S8. Comparative analysis of products formed by PARP1 and PARP2. The automodification of PARP1/PARP2 was performed in the reaction conditions of DLS measurements (for details see Supplementary Methods). Gel image (A) and kinetic curve (B) show formation of PARylated PARP1 and PARP2; the modification products after 15-min incubation were subjected to PARG treatment (A, lane 6 for PARP1 and 12 for PARP2). Positions of unmodified and PARylated PARP1/PARP2 and of molecular weight protein markers are indicated on the gel image. The alkaline hydrolysis analysis of the length of PAR polymer synthesized by PARP1 and PARP2 after 15-min incubation is presented in panel C.

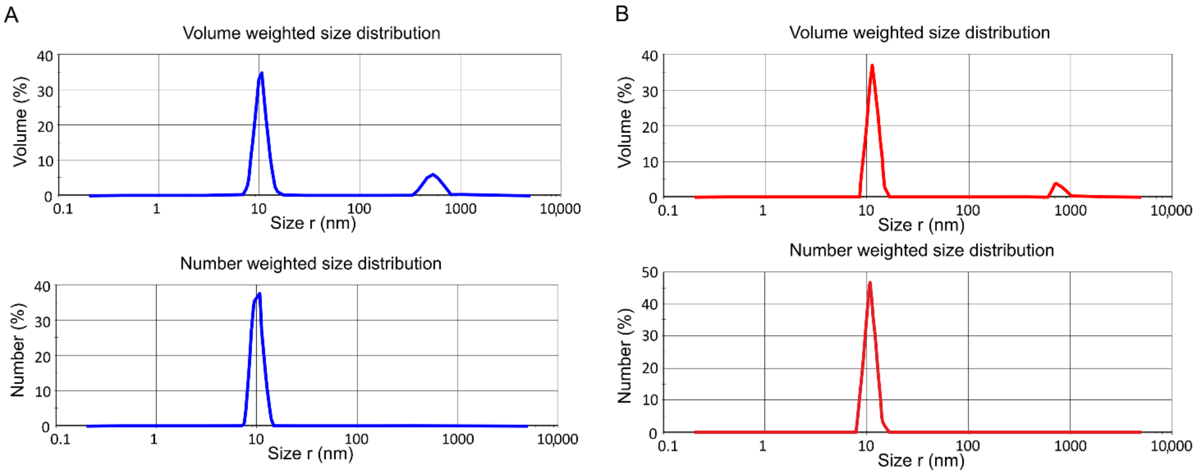

Figure S9. Volume- and number-weighted size distribution profiles for PARylated PARP2–XRCC1 (A) and PARP2–PARP1 (B) protein mixtures. Peaks of huge associates with R_H value of 550 nm for the PARP2-XRCC1 and 750 nm for the PARP2–PARP1 mixture were detected only in the volume-weighted size distribution profiles.

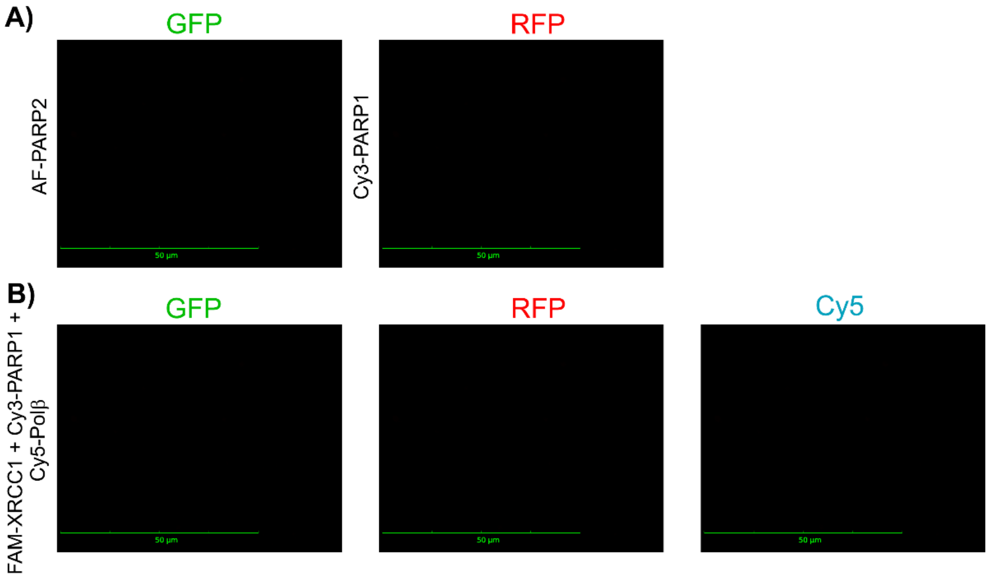

Figure S10. Fluorescence detection of associates formed upon PARylation reaction catalysed by PARP1/PARP2 (A) or PARP1 in the presence of XRCC1 and Polβ (B) after incubation of samples with EDTA. The proteins labelled with distinct fluorophores (AF-PARP2, Cy3-PARP1, FAM-XRCC1, Cy5-Polβ) were visualized in images acquired with appropriate filters (GFP for AF, FAM; RFP for Cy3; Cy5 for Cy5). Scale bar, 50 µM. Preparation of samples is detailed in Materials and methods (main text).

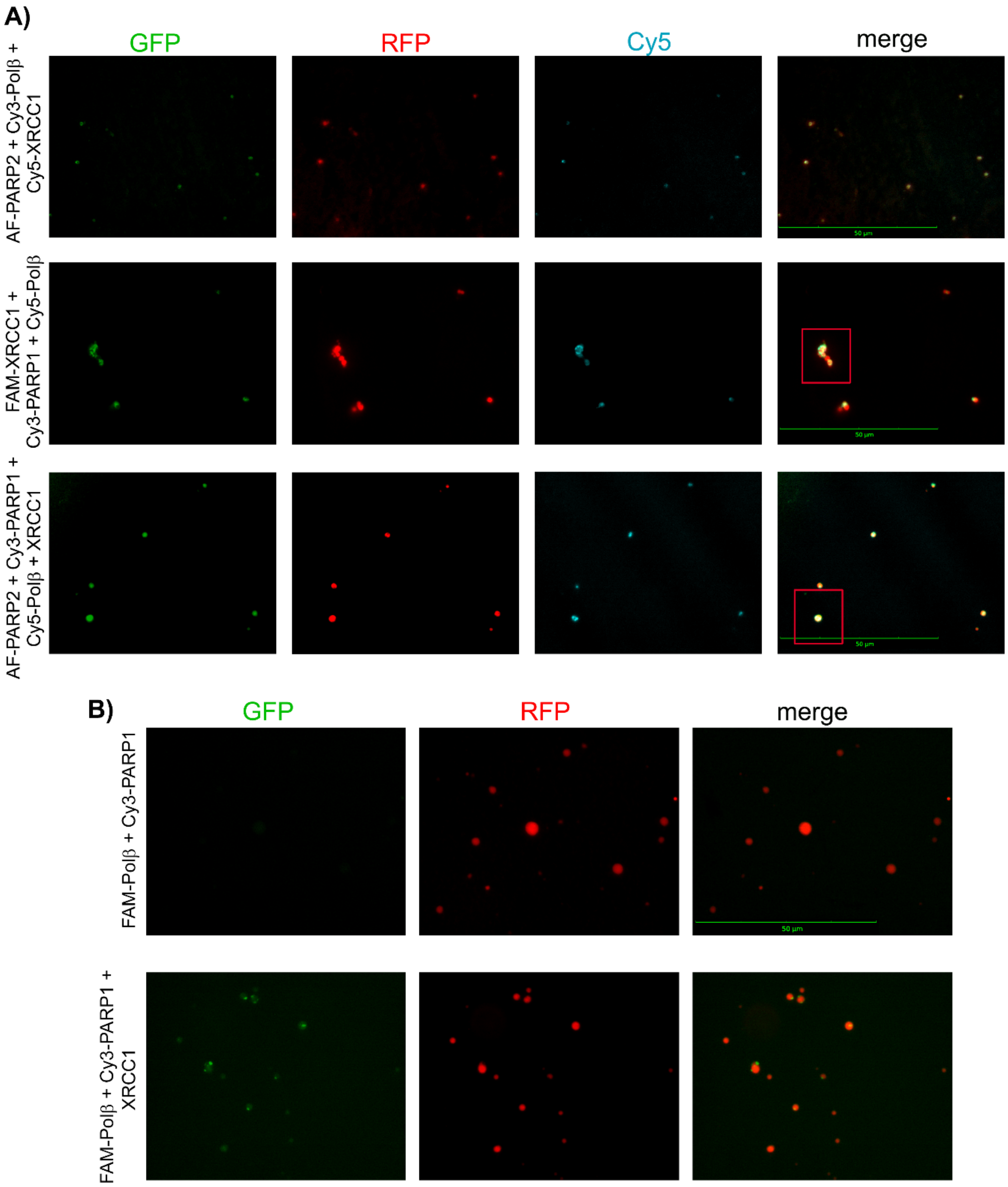

Figure S11. (A) Fluorescence detection of associates formed upon PARylation reaction catalysed by PARP1/PARP2 or their equimolar mixture, in the presence or absence of XRCC1 and Polβ. The proteins labelled with distinct fluorophores (as specified from the left of image series) were visualized in images acquired with appropriate filters (indicated above the images); merge images show colocalization of proteins labelled with distinct fluorophores. The enlarged images of selected merged areas are presented in Figure 5 in the main text. (B) Visualization of FAM-Polβ and its colocalization with associates formed upon PARylation reaction catalysed by Cy3-labelled PARP1 depends on the presence of XRCC1.

Supplementary Tables

Table S1. Labelling of PARP2, PARP1, XRCC1, APE1 and Polβ with various fluorescent probes.

Protein	Reagent [1]	Molar ratio reagent:protein [2]	Stoichiometry of labelling, mol of dye / mol of protein
PARP2	FAM-SE	3:1	0.60
PARP2	AF488-SE	3:1	0.50
PARP1	Cy3-SE	3:1	0.65
PARP1	FAM-SE	3:1	0.98
XRCC1	Cy3-SE	3:1	1.02
XRCC1	Cy5-SE	3:1	1.05
Polβ	FAM-SE	1.6:1	0.76
Polβ	AF488-SE	1.6:1	0.25
Polβ	Cy3-SE	1.6:1	0.98
Polβ	Cy5-SE	1.6:1	1.02
APE	FAM-SE	1.6:1	0.86

Copyright: © 2021 by the authors. Licensee MDPI, Basel, Switzerland. This article is an open access article distributed under the terms and conditions of the Creative Commons Attribution (CC BY) license (http://creativecommons.org/licenses/by/4.0/).

[1] FAM-SE, AF488-SE, Cy3-SE, Cy5-SE – N-succinimidyl ester of 5(6)-carboxyfluorescein, 5-AF488, sulfo-Cyanine 3, or sulfo-Cyanine 5. [2] The reaction mixture contained 100 mM MES, pH 7.0, 150 mM NaCl, 80-100 μM protein and respective excessive concentration of the reactive probe.

Table S2. Experimentally determined average R_H values of PARP2 and its heterocomplexes with BER proteins.

Protein(s) [1]	R_H [2] (nm) from size distributions weighted by		
	intensity	volume	number
	Homo-oligomerization		
PARP2	5.4 ± 0.4	5.0 ± 0.4	4.6 ± 0.3
	Hetero-oligomerization		
PARP2 + Polβ	6.0 ± 0.1	5.6 ± 0.3	5.4 ± 0.2
PARP2 + APE1	5.2 ± 0.1	4.9 ± 0.2	4.7 ± 0.1
PARP2 + XRCC1	9.2 ± 0.4	7.6 ± 0.3	6.5 ± 0.3
PARP2 + PARP1	9.3 ± 0.5	7.8 ± 0.4	6.9 ± 0.3

[1] The concentration of proteins (individual and in the equimolar mixtures) was 6 μM. [2] Values are the mean (± SD) of three independent experiments.

Table S3. Analysis of PARylated PARP1 by DLS. [1]

Protein(s) [2]	R_H [3], (nm)			
	**Before reaction initiation**	**PARylated protein associate [4]**	**PARylated protein after addition of EDTA**	**PARylated protein after PARG treatment**
PARP1	8.0 ± 0.5	1000 ± 56	21.6 ± 0.4	8.2 ± 0.4
PARP1 + Polβ	7.1 ± 0.3	1015 ± 60	19.4 ± 0.7	8.4 ± 0.3
PARP1 + APE1	6.9 ± 0.2	1021 ± 56	18.1 ± 0.5	8.3 ± 0.2
PARP1 + XRCC1	9.8 ± 0.9	900 ± 40	21.9 ± 0.7	10.4 ± 0.6

[1] Data from our previous study [2] are presented for comparison with present data for PARP2 (Table 4, main text). [2] The concentration of proteins (individual and in the equimolar mixtures) was 6 μM. [3] Experimentally determined average R_H value of species in single peaks from volume weighted size distribution. Values are the mean (± SD) of three independent experiments. [4] Poly(ADP-ribose) synthesis was performed in the presence of Mg^{2+} for 15-min incubation.

References

1. Moor, N.A.; Vasil'eva, I.A.; Anarbaev, R.O.; Antson, A.A.; Lavrik, O.I. Quantitative characterization of protein-protein complexes involved in base excision DNA repair. *Nucleic Acids Res.* **2015**, *43*, 6009–6022. doi: 10.1093/nar/gkv569.
2. Marintchev, A.; Robertson, A.; Dimitriadis, E.K.; Prasad, R.; Wilson, S.H.; Mullen, G.P. Domain specific interaction in the XRCC1-DNA polymerase β complex, *Nucleic Acids Res.* **2000**, *28*, 2049–2059. doi: 10.1093/nar/28.10.2049.
